# Novel Information Measures for Fermatean Fuzzy Sets and Their Applications to Pattern Recognition and Medical Diagnosis

**DOI:** 10.1155/2023/9273239

**Published:** 2023-03-08

**Authors:** Shahzaib Ashraf, Attaullah, Muhammad Naeem, Asghar Khan, Noor Rehman, M. K. Pandit

**Affiliations:** ^1^Institute of Mathematics, Khwaja Fareed University of Engineering and Information Technology, Rahim Yar Khan 64200, Pakistan; ^2^Department of Mathematics, Abdul Wali Khan University, Mardan 23200, Pakistan; ^3^Department of Mathematics, Deanship of Applied Sciences, Umm Al-Qura University, Makkah 24382, Saudi Arabia; ^4^Department of Mathematics, Bacha Khan University, Charsadda 24420, Pakistan; ^5^Department of Mathematics, Jahangirnagar University, Savar, Dhaka, Bangladesh

## Abstract

Fermatean fuzzy sets (FFSs) have piqued the interest of researchers in a wide range of domains. The striking framework of the FFS is keen to provide the larger preference domain for the modeling of ambiguous information deploying the degrees of membership and nonmembership. Furthermore, FFSs prevail over the theories of intuitionistic fuzzy sets and Pythagorean fuzzy sets owing to their broader space, adjustable parameter, flexible structure, and influential design. The information measures, being a significant part of the literature, are crucial and beneficial tools that are widely applied in decision-making, data mining, medical diagnosis, and pattern recognition. This paper aims to expand the literature on FFSs by proposing many innovative Fermatean fuzzy sets-based information measures, namely, distance measure, similarity measure, entropy measure, and inclusion measure. We investigate the relationship between distance, similarity, entropy, and inclusion measures for FFSs. Another achievement of this research is to establish a systematic transformation of information measures (distance measure, similarity measure, entropy measure, and inclusion measure) for the FFSs. To accomplish this aim, new formulae for information measures of FFSs have been presented. To demonstrate the validity of the measures, we employ them in pattern recognition, building materials, and medical diagnosis. Additionally, a comparison between traditional and novel similarity measures is described in terms of counter-intuitive cases. The findings demonstrate that the innovative information measures do not include any absurd cases.

## 1. Introduction

The idea of the fuzzy set (FS) was developed by Zadeh [1] in 1965, which addressed vagueness and ambiguity in real-world situations. In 1970, Bellman and Zadeh (1970) introduced the concept of decision-making (DM) problems with uncertainty. DM is a systematic procedure of selecting the most ideal choice from a collection of available alternatives. Therefore, the decision maker plays a crucial role in real world environments [2]. A smart decision may have a significant impact on the direction of someone's lifestyle. Before making a final selection, a DM assesses the restrictions, advantages, and characteristics of each alternative. Since an FS is defined by a single parameter: membership degree. Several higher-order FSs have been described in recent decades by several scholars.

Atanassov [3] established the notion of intuitionistic fuzzy sets (IFSs) capable of dealing with complexity and uncertainty and it has been extensively examined and utilized by several researchers in DM problems. An IFS is defined by three parameters: membership grade (MG), nonmembership grade (NMG), and hesitancy margin with the property that the sum of MG and NMG must be less than or equal to 1.

In many situations, it is conceivable that the sum of the MG and NMG will be greater than 1. To overcome these challenges, Yager [4] introduced the Pythagorean fuzzy set (PyFS) as an extension of the IFS theory. PyFS is defined by an MG and NMG and satisfies the criterion that the square sum of its MG and NMG is less than or equal to 1. Therefore, PyFSs can more accurately express the fuzzy nature of information than IFS.

In the field of PyFS, there are various approaches for solving real-life multiattribute decision-making (MADM) situations. A number of researchers have also suggested real-world applications in a Pythagorean fuzzy environment. However, if orthopair FSs as 〈0.9,0.5〉, where 0.9 is the MG of specific criteria of a parameter and 0.5 is the NMG, it does not fulfill the IFS and PFS requirements. However, the cubic sum of the MG and NMG is equal to or less than one. In this context, Senapati and Yager [5] recently introduced the Fermatean fuzzy set (FFS). They also demonstrated that FFSs have larger degrees of uncertainty than IFSs and PyFSs, are capable of sustaining higher levels of uncertainty, and can solve MCDM challenges. Information measures are an essential notion for dealing with MADM challenges in a variety of domains, including pattern recognition, clinical diagnosis, and personnel appointment. There are several types of information measures established such as distance, similarity, entropy, and inclusion measures.

The MADM process are normally assisted by similarity measures, distance measures, inclusion measures, entropy measures, and, in certain situations, aggregation operators. The degree of similarity measures has garnered considerable interest in recent decades due to its importance in DM, data mining, pattern recognition, and medical diagnosis applications. Szmidt and Kacprzyk [6] performed the first investigation, extending well-known distance measures such as the Hamming distance and the Euclidian distance to the IFS environment and comparing them to approaches used for conventional fuzzy sets. However, Wang and Xin [7] suggested that Szmidt and Kacprzyk [6] distance measure was ineffective in certain situations. Therefore, several innovative pattern recognition distance measures were developed and implemented. Grzegorzewski [8] also extended Hamming, Euclidean, and their normalized versions to the IFS framework. Later on, Chen [9] demonstrated that several flaws occurred in Grzegorzewski [8] by providing counter-examples. Hung and Yang [10] described three similarity measures and extended the Hausdorff distance to IFSs. On the other side, rather than expanding well-established measures, various research established novel similarity measures for IFS.

Yong et al. [11] developed a novel similarity measure for IFS based on MG and NMG. Mitchell [12] demonstrated that Yong et al.'s [11] similarity measure had certain counter-intuitive circumstances and improved it statistically. Additionally, Liang and Shi [13] provided examples to demonstrate that the similarity measure proposed by Yong et al. [11] was unsuitable for certain scenarios, and hence developed various additional similarity measures for IFSs.

Xu [14] formulated a series of IFS-based similarity measures and applied them to the MADM problem employing IF information. Xu and Chen [15] presented a set of distance and similarity measures that are different combinations and extensions of the weighted Hamming, Euclidean, and Hausdorff distances. Xu and Yager [16] constructed a similarity measure between IFSs and used it to MAGDM utilizing IF preference relations.

In addition to this research, several researchers investigated the relationships between IFSs' distance, similarity, and entropy measures. Zeng and Guo [17] analyzed the relationship between normalized distance, similarity, inclusion, and entropy of interval-valued fuzzy collections. Additionally, it was demonstrated that the similarity, inclusion, and entropy of interval-valued fuzzy sets may be induced using the normalized distance of their axiomatic definitions. Wei et al. [18] proposed a generalized entropy measure for IFSs and PyFSs. Additionally, a technique was developed for constructing similarity measures for IFS and PyFSs using entropy measures. Numerous researchers investigated information measures (distance measure, similarity measure, entropy measure, and inclusion measure) for IFSs and PyFSs and their transformations relationship. Dengfeng and Chuntian [19] investigated the similarity between IFSs and used their findings to pattern recognition. Huang and Yang [10] presented the Hausdorff distance as a similarity measure between IFSs and utilized it to assess the degree of similarity between IFSs. Ashraf et al. [20] gave the idea of a spherical fuzzy set then they implicated this concept also in decision-making [21].

Nguyen et al. [22] developed a novel knowledge-based similarity measure for IFSs and demonstrated its application to pattern recognition. Zhang [23] pioneered a unique strategy for PyFSs MADM based on similarity measures. Zhang et al. [24] explored the use of the application of a scoring function on IFSs with double parameters for pattern recognition and medical diagnosis. Ejegwa established distance [25] and similarity measures [26] for PyFSs.

Ye [27] designed and implemented a cosine similarity measure for IFSs (CIFS). In addition, Ye [28] introduced the cosine similarity measure for interval-valued IFSs (CIVIFSs) and described its use in solving MADM problems. Liu et al. [29] investigated the cosine similarity measure between hybrid IFSs and their application for diagnostic purposes. In recent years, several scholars have conducted research on PyFS information measures (distance measure, similarity measure, entropy measure, and inclusion measure). Wei and Wei [30] introduced a set of ten cosine-based PFS similarity measures relying on the MG, NMG, and hesitation of PyFSs in order to improve the capacity to cope with the two optimization challenges related to pattern recognition and medical diagnosis procedures. Peng [31] established a PyFS similarity measure based on the parameters Lp norm and levels of ambiguity, which were examined in detail in relation to the PyFS similarity measure. Peng et al. [32] developed the fundamental definitions of PyFS information measures, along with the similarity measure, as well as discussed the transformation principles for the established information measures.

The advantages of existing information measures are as follows:

An examination of the existing literature on FS, IFS, and PyFS exposes a number of weaknesses that spur us to create a more potent class of novel information measures (distance measure, similarity measure, entropy measure, and inclusion measure).

The disadvantages of existing information measures are as follows:

- Some of them cannot help but be caught in pointless circumstances (i.e., dividing by zero).
- Many of them struggle to avoid examples that seem to go against logic.
- Many of them are unable to categorize the results, and some of them provide irrational results. We describe a class of useful FFS information measures (distance measure, similarity measure, entropy measure, and inclusion measure), offer associated information measure formulations, and examine their transformation connections to address the flaw in the prior research.

The important contributions of the current manuscript are listed.

### 1.1. Important Contributions of the Manuscript

- Development of axiomatic FFS information measures (distance measure, similarity measure, entropy, and inclusion measure).
- We develop several equations for FFS information metrics and thoroughly examine the relevant transformation relationships.
- To determine the necessary distances for pattern recognition and medical diagnostics in order to show that they are effective, we compared the suggested FFS distance measurements to those that are previously documented in the literature. Moreover, we demonstrate the viability and efficiency of using distance measurements between FFS data.
- A number of illogical examples of traditional similarity measures are used to show the effectiveness of the novel measures.
- We use them for pattern recognition, building supplies, and medical diagnosis.
- We come to the conclusion that the proposed similarity measures outperform current similarity measures for pattern recognition problems.
- To show the effectiveness of the suggested information measures, which are also contrasted with current similarity measures, numerical examples are offered. For issues with medical diagnosis, a comparison between the proposed similarity measures and traditional similarity measurements is carried out.
- Additionally, we used an example to demonstrate how the suggested FFS inclusion measures could be used for pattern identification. The results show that new illustration approaches are both feasible and successful.

The manuscript is organized as follows: Section 2 discusses the definitions and fundamental ideas of FS, HFS, IFS, and FFS, as well as the corresponding operational rules of FFS.  Section 3 introduces a new type of information measures, provides related information measure formulations, and investigates their transformation relationships for FFSs. In Sections 4to 7, we demonstrated the application of the novel information measures between FFSs to pattern recognition. Moreover, a comparative study has been presented between the proposed similarity measure and conventional similarity measures.  Section 7 concludes the paper by outlining the future area of research.

## 2. Basic Terminologies

In this section, we provide some relevant fundamental information, such as FS, HFS, IFS, FFSs, and some related operational laws, which are listed. These core concepts will assist readers in comprehending the proposed framework.

**Definition 1 .**

(see [1]). Let *𝒵*={ϰ_1_, ϰ_2_, ϰ_3_,…, ϰ_*n*_} be a finite set. The FS *F* on *𝒵* is defined as follows:

(1) $$\begin{array}{c} F=\left\{\langle \varkappa_{i},I_{F}\left(\varkappa_{i}\right)\rangle |\varkappa_{i}\in Z\right\}\text{,} \end{array}$$

where *F*(ϰ_*i*_) ∈ [0,1] known to be MG.

**Definition 2 .**

(see [3]). Let *𝒵*={ϰ_1_, ϰ_2_, ϰ_3_,…, ϰ_*n*_} be a finite set. An IFS *Ϝ* over ϰ is follows:

(2) $$\begin{array}{c} Ϝ=\left\{\langle \varkappa_{i},I_{Ϝ}\left(\varkappa_{i}\right),{ð}_{Ϝ}\left(\varkappa_{i}\right)\rangle |\varkappa_{i}\in Z\right\}\text{,} \end{array}$$

for each ϰ_*i*_ ∈ *𝒵* the functions *ℑ*_*Ϝ*_ : *𝒵*⟶[0, 1], 1 and *ð*_*Ϝ*_ : ϰ⟶[0, 1] denotes the MG and NMG, respectively, which must satisfy the property 0 ≤ *ℑ*_*Ϝ*_(ϰ_*i*_)+ð_*Ϝ*_(ϰ_*i*_) ≤ 1.

**Definition 3 .**

(see [33]). Let ϰ={ϰ_1_, ϰ_2_, ϰ_3_,…, ϰ_*n*_} be a finite set. The HFS *H* on ϰ is defined as follows:

(3) $$\begin{array}{c} H=\left\{\langle \varkappa_{i},I_{h_{H}}\left(\varkappa_{i}\right)\rangle |\varkappa_{i}\in Z\right\}\text{,} \end{array}$$

where *h*(ϰ_*i*_) is a set of values contain in (0,1), which shows the MG of ϰ_*i*_ ∈ *H*. The element of *h*(ϰ_*i*_) is known as the HF element.

**Definition 4 .**

(see [34]). Let *𝒵*={ϰ_1_, ϰ_2_, ϰ_3_,…, ϰ_*n*_} be a finite set, a Fermatean fuzzy sets (FFSs) *ℱ* over *𝒵* is defined as follows:

(4) $$\begin{array}{c} F=\left\{\langle \varkappa_{i},I_{F}\left(\varkappa_{i}\right),{ð}_{F}\left(\varkappa_{i}\right)\rangle |\varkappa_{i}\in \varkappa \right\}\text{,} \end{array}$$

for each ϰ_*i*_ ∈ ϰ the functions *ℑ*_*ℱ*_ : ϰ⟶[0, 1] and ð_*ℱ*_ : ϰ⟶[0, 1] denote the MG and NMG, respectively, which must satisfy (ð_*ℱ*_(ϰ_*i*_))^3^+(*ℑ*_*ℱ*_(ϰ_*i*_))^3^ ≤ 1. The degree of indeterminacy is given as follows:

(5) $$\begin{array}{c} \pi_{F}\left(\varkappa_{i}\right)=\sqrt[{3}]{1-{\left(I_{F}\left(\varkappa_{i}\right)\right)}^{3}+{\left({ð}_{F}\left(\varkappa_{i}\right)\right)}^{3}}\text{.} \end{array}$$

**Definition 5 .**

(see [5]). If *𝒴* and *𝒫* be two FFSs, then the operations can be defined as follows:

- Addition: $𝒴⊕𝒫=\left\{\varkappa_{i}\in 𝒵,\sqrt[{3}]{{ℑ}_{𝒴}^{3}\left(\varkappa_{i}\right)+{ℑ}_{𝒫}^{3}\left(\varkappa_{i}\right)-{ℑ}_{𝒴}^{3}\left(\varkappa_{i}\right){ℑ}_{𝒫}^{3}\left(\varkappa_{i}\right)},{ð}_{𝒴}\left(\varkappa_{i}\right){ð}_{𝒫}\left(\varkappa_{i}\right)|\varkappa_{i}\in 𝒵\right\}$
- Multiplication: $𝒴⊗𝒫=\left\{\varkappa_{i}\in 𝒵,{ℑ}_{𝒴}\left(\varkappa_{i}\right){ℑ}_{𝒫}\left(\varkappa_{i}\right),\sqrt[{3}]{{ð}_{𝒴}^{3}\left(\varkappa_{i}\right)+{ð}_{𝒫}^{3}\left(\varkappa_{i}\right)-{ð}_{𝒴}^{3}\left(\varkappa_{i}\right){ð}_{𝒫}^{3}\left(\varkappa_{i}\right)}|\varkappa_{i}\in 𝒵\right\}$
- Scalar multiplication: $\lambda ⊙𝒴=\left\{\varkappa_{i}\in 𝒵,\sqrt[{3}]{1-{\left(1-{ℑ}_{𝒴}^{3}\left(\varkappa_{i}\right)\right)}^{\lambda },{\left({ð}_{𝒴}^{3}\left(\varkappa_{i}\right)\right)}^{\lambda }}\left|\varkappa_{i}\right.\in 𝒵\;\text{and}\;\lambda 0\right\}$
- Exponent: ${𝒴}^{\lambda }=\left\{\varkappa_{i}\in 𝒵,{ℑ}_{𝒴}^{3}\left(\varkappa_{i}\right),\sqrt[{3}]{1-{\left(1-{ð}_{𝒫}^{3}\left(\varkappa_{i}\right)\right)}^{\lambda }}\left|\varkappa_{i}\right.\in 𝒵\right\}$

**Definition 6 .**

(see [5]). If *𝒴* and *𝒫* be two FFSs, then the operations can be defined as follows:

- Complement: *𝒴*^*c*^={〈ð_*𝒴*_(ϰ_*i*_), *ℑ*_*𝒴*_(ϰ_*i*_)〉*|*ϰ_*i*_ ∈ *𝒵*};
- Equality: *𝒴*=*𝒫* iff for all 〈ϰ_*i*_ ∈ *𝒵*, *ℑ*_*𝒴*_(ϰ_*i*_)=*ℑ*_*𝒫*_(ϰ_*i*_)and ð_*𝒴*_(ϰ_*i*_)=ð_*𝒫*_(ϰ_*i*_)〉;
- Intersection: *𝒴*∩*𝒫*=〈min (*ℑ*_*𝒴*_(ϰ_*i*_), *ℑ*_*𝒫*_(ϰ_*i*_)), max (ð_*𝒴*_(ϰ_*i*_), ð_*𝒫*_(ϰ_*i*_))*|*ϰ_*i*_ ∈ *𝒵*〉;
- Union: *𝒴* ∪ *𝒫*=〈max (*ℑ*_*𝒴*_(ϰ_*i*_), *ℑ*_*𝒫*_(ϰ_*i*_)), min (ð_*𝒴*_(ϰ_*i*_), ð_*𝒫*_(ϰ_*i*_))*|*ϰ_*i*_ ∈ *𝒵*〉.

**Definition 7 .**

(see [5]). If *𝒴*, *𝒫*, and *ℳ* be three FFSs, then the following characteristics are held:

- *𝒴* ∪ *𝒫*=*𝒫* ∪ *𝒴*;
- *𝒴*∩*𝒫*=*𝒫*∩*𝒴*;
- *𝒴* ∪ (*𝒫* ∪ *ℳ*)=(*𝒴* ∪ *𝒫*) ∪ *ℳ*;
- *𝒴*∩(*𝒫*∩*ℳ*)=(*𝒴*∩*𝒫*)∩*ℳ*;
- *α* ∪ (*𝒴* ∪ *𝒫*)=*α𝒴* ∪ *α𝒫*;
- (*𝒴* ∪ *𝒫*)^*α*^=*𝒴*^*α*^ ∪ *𝒫*^*α*^.

## 3. Some New Types of Information Measures between FFSs

This section explains the axiomatic framework of FFSs information measures (distance, similarity, entropy, and inclusion), as well as their related formulations. Simultaneously, their transformation relationships are thoroughly examined.

### 3.1. Distance Measures for FFSs

This section introduces the idea of a distance measures for FFSs. A number that is assigned to a pair of points in a space which indicates how far those points are from one another. A distance measure is called a metric if it is always positive and also it is always symmetric.

**Definition 8 .**

Let *𝒴*, *𝒫*, and *ℳ* be three FFSs on *𝒵*. A distance measure *𝒟*(*𝒴*, *𝒫*) is a mapping *𝒟*: FFS (*𝒵*)× FFS (*𝒵*)⟶[0,1], carrying the following features:

1. 0 ≤ *𝒟*(*𝒴*, *𝒫*) ≤ 1;
2. *𝒟*(*𝒴*, *𝒫*)=*𝒟*(*𝒫*, *𝒴*);
3. *𝒟*(*𝒴*, *𝒫*)=0 iff *𝒴*=*𝒫*;
4. *𝒟*(*𝒴*, *𝒴*^*c*^)=1 iff *𝒴* is a crisp set;
5. If *𝒴*⊆*𝒫*⊆*ℳ*, then *𝒟*(*𝒴*, *𝒫*) ≤ *𝒟*(*𝒴*, *ℳ*) and *𝒟*(*𝒫*, *ℳ*) ≤ *𝒟*(*𝒴*, *ℳ*).

**Theorem 1 .**

Let *𝒴* and *𝒫* be two FFS *s*, then *𝒟*_*i*_(*𝒴*, *𝒫*)(*i*=1,2,…, 13) is a distance measure.

1. *𝒟*(*𝒴*, *𝒫*)=(1/2|*𝒵*|)∑_ϰ_*i*_∈*𝒵*_(|*ℑ*_*𝒴*_^3^(ϰ_*i*_) − *ℑ*_*𝒫*_^3^(ϰ_*i*_)|+|ð_*𝒴*_^3^(ϰ_*i*_) − ð_*𝒫*_^3^(ϰ_*i*_)|+|*π*_*𝒴*_^3^(ϰ_*i*_) − *π*_*𝒫*_^3^(ϰ_*i*_)|);
2. *𝒟*_2_(*𝒴*, *𝒫*)=(1/2|*𝒵*|)∑_ϰ_*i*_∈*𝒵*_(|(*ℑ*_*𝒴*_^3^(ϰ_*i*_) − *ℑ*_*𝒫*_^3^(ϰ_*i*_))+(ð_*𝒴*_^3^(ϰ_*i*_) − ð_*𝒫*_^3^(ϰ_*i*_))|);
3. *𝒟*_3_(*𝒴*, *𝒫*)=(1/4|*X*|)(∑_ϰ_*i*_∈*𝒵*_(|*ℑ*_*𝒴*_^3^(ϰ_*i*_) − *ℑ*_*𝒫*_^3^(ϰ_*i*_)|+|ð_*𝒴*_^3^(ϰ_*i*_) − ð_*𝒫*_^3^(ϰ_*i*_)|+|*π*_*𝒴*_^3^(ϰ_*i*_) − *π*_*𝒫*_^3^(ϰ_*i*_)|)+∑_ϰ_*i*_∈*𝒵*_(|(*ℑ*_*𝒴*_^3^(ϰ_*i*_) − ð_*𝒴*_^3^(ϰ_*i*_)) − (*ℑ*_*𝒫*_^3^(ϰ_*i*_) − ð_*𝒫*_^3^(ϰ_*i*_))|))
4. *𝒟*_4_(*𝒴*, *𝒫*)=(1/|*𝒵*|)∑_ϰ_*i*_∈*𝒵*_(|*ℑ*_*𝒴*_^3^(ϰ_*i*_) − *ℑ*_*𝒫*_^3^(ϰ_*i*_)|∨|(ð_*𝒴*_^3^(ϰ_*i*_) − ð_*𝒫*_^3^(ϰ_*i*_))|);
5. *𝒟*_5_(*𝒴*, *𝒫*)=(2/|*𝒵*|)∑_ϰ_*i*_∈*𝒵*_(|*ℑ*_*𝒴*_^3^(ϰ_*i*_) − *ℑ*_*𝒫*_^3^(ϰ_*i*_)|∨|(ð_*𝒴*_^3^(ϰ_*i*_) − ð_*𝒫*_^3^(ϰ_*i*_))|/1+|*ℑ*_*𝒴*_^3^(ϰ_*i*_) − *ℑ*_*𝒫*_^3^(ϰ_*i*_)|∨|(ð_*𝒴*_^3^(ϰ_*i*_) − ð_*𝒫*_^3^(ϰ_*i*_))|);
6. *𝒟*_6_(*𝒴*, *𝒫*)=(2∑_ϰ_*i*_∈*𝒵*_|*ℑ*_*𝒴*_^3^(ϰ_*i*_) − *ℑ*_*𝒫*_^3^(ϰ_*i*_)|∨|(ð_*𝒴*_^3^(ϰ_*i*_) − ð_*𝒫*_^3^(ϰ_*i*_))|/∑_ϰ_*i*_∈*𝒵*_(1+|*ℑ*_*𝒴*_^3^(ϰ_*i*_) − *ℑ*_*𝒫*_^3^(ϰ_*i*_)|∨|ð_*𝒴*_^3^(ϰ_*i*_) − ð_*𝒫*_^3^(ϰ_*i*_)|));
7. *𝒟*_7_(*𝒴*, *𝒫*)=1 − *α*(∑_ϰ_*i*_∈*𝒵*_(*ℑ*_*𝒴*_^3^(ϰ_*i*_)∧*ℑ*_*𝒫*_^3^(ϰ_*i*_))/∑_ϰ_*i*_∈*𝒵*_(*ℑ*_*𝒴*_^3^(ϰ_*i*_)∨*ℑ*_*𝒫*_^3^(ϰ_*i*_))) − *β*(∑_ϰ_*i*_∈*𝒵*_(ð_*𝒴*_^3^(ϰ_*i*_)∧ð_*𝒫*_^3^(ϰ_*i*_))/∑_ϰ_*i*_∈*𝒵*_(ð_*𝒴*_^3^(ϰ_*i*_)∨ð_*𝒫*_^3^(ϰ_*i*_))), *α*+*β*=1, *α*, *β* ∈ [0,1];
8. *𝒟*_8_(*𝒴*, *𝒫*)=1 − (*α*/|*𝒵*|)∑_ϰ_*i*_∈*𝒵*_(*ℑ*_*𝒴*_^3^(ϰ_*i*_)∧*ℑ*_*𝒫*_^3^(ϰ_*i*_))/(*ℑ*_*𝒴*_^3^(ϰ_*i*_)∨*ℑ*_*𝒫*_^3^(ϰ_*i*_)) − (*β*/|*𝒵*|)∑_ϰ_*i*_∈*𝒵*_(ð_*𝒴*_^3^(ϰ_*i*_)∧ð_*𝒫*_^3^(ϰ_*i*_))/(ð_*𝒴*_^3^(ϰ_*i*_)∨ð_*𝒫*_^3^(ϰ_*i*_)), *α*+*β*=1, *α*, *β* ∈ [0,1];
9. *𝒟*_9_(*𝒴*, *𝒫*)=1 − (1/|*𝒵*|)∑_ϰ_*i*_∈*𝒵*_((*ℑ*_*𝒴*_^3^(ϰ_*i*_)∧*ℑ*_*𝒫*_^3^(ϰ_*i*_))+(ð_*𝒴*_^3^(ϰ_*i*_)∧ð_*𝒫*_^3^(ϰ_*i*_))/(*ℑ*_*𝒴*_^3^(ϰ_*i*_)∨*ℑ*_*𝒫*_^3^(ϰ_*i*_))+(ð_*𝒴*_^3^(ϰ_*i*_)∨ð_*𝒫*_^3^(ϰ_*i*_)));
10. *𝒟*_10_(*𝒴*, *𝒫*)=1 − (∑_ϰ_*i*_∈*𝒵*_(*ℑ*_*𝒴*_^3^(ϰ_*i*_)∧*ℑ*_*𝒫*_^3^(ϰ_*i*_))+(ð_*𝒴*_^3^(ϰ_*i*_)∧ð_*𝒫*_^3^(ϰ_*i*_))/∑_ϰ_*i*_∈*𝒵*_(*ℑ*_*𝒴*_^3^(ϰ_*i*_)∨*ℑ*_*𝒫*_^3^(ϰ_*i*_))+(ð_*𝒴*_^3^(ϰ_*i*_)∨ð_*𝒫*_^3^(ϰ_*i*_)));
11. *𝒟*_11_(*𝒴*, *𝒫*)=1 − (1/|*𝒵*|)∑_ϰ_*i*_∈*𝒵*_((*ℑ*_*𝒴*_^3^(ϰ_*i*_)∧*ℑ*_*𝒫*_^3^(ϰ_*i*_))+(1 − ð_*𝒴*_^3^(ϰ_*i*_))∧(1 − ð_*𝒫*_^3^(ϰ_*i*_)))/((*ℑ*_*𝒴*_^3^(ϰ_*i*_)∨*ℑ*_*𝒫*_^3^(ϰ_*i*_))+(1 − ð_*𝒴*_^3^(ϰ_*i*_))∨(1 − ð_*𝒫*_^3^(ϰ_*i*_)));
12. *𝒟*_12_(*𝒴*, *𝒫*)=1 − (∑_ϰ_*i*_∈*𝒵*_(*ℑ*_*𝒴*_^3^(ϰ_*i*_)∧*ℑ*_*𝒫*_^3^(ϰ_*i*_))+((1 − ð_*𝒴*_^3^(ϰ_*i*_))∧(1 − ð_*𝒫*_^3^(ϰ_*i*_))))/(∑_ϰ_*i*_∈*𝒵*_(*ℑ*_*𝒴*_^3^(ϰ_*i*_)∨*ℑ*_*𝒫*_^3^(ϰ_*i*_))+((1 − ð_*𝒴*_^3^(ϰ_*i*_))∨(1 − ð_*𝒫*_^3^(ϰ_*i*_))));
13. ${𝒟}_{13}\left(𝒴,𝒫\right)=\sqrt[{t}]{\begin{array}{c} \left(1/2\left|𝒵\right|{\left(l_{1}+1\right)}^{t}\right)\sum_{\varkappa_{i}\in 𝒵}\left\{{\left|\left(l_{1}\left({ℑ}_{𝒴}^{3}\left(\varkappa_{i}\right)\right)-{ℑ}_{𝒫}^{3}\left(\varkappa_{i}\right)\right)-\left({ð}_{𝒴}^{3}\left(\varkappa_{i}\right)-{ð}_{𝒫}^{3}\left(\varkappa_{i}\right)\right)\right|}^{t}\right\}\\ +\left(1/2\left|𝒵\right|{\left(l_{2}+1\right)}^{t}\right)\sum_{\varkappa_{i}\in 𝒵}\left\{{\left|\left(l_{2}\left({ð}_{𝒴}^{3}\left(\varkappa_{i}\right)\right)-{ð}_{𝒫}^{3}\left(\varkappa_{i}\right)\right)-\left({ℑ}_{𝒴}^{3}\left(\varkappa_{i}\right)-{ℑ}_{𝒫}^{3}\left(\varkappa_{i}\right)\right)\right|}^{t}\right\} \end{array}}$

### 3.2. Similarity Measure for FFSs

This section introduces the idea of similarity measures for FFSs. Similarity functions take a pair of points and return a large similarity value for nearby points, and a small similarity value for distant points. One way to transform between a distance function and a similarity measure is to take the reciprocal.

**Definition 9 .**

Let *𝒴*, *𝒫*, and *ℳ* be three FFSs on *𝒵*. A similarity measure *𝒮*(*𝒴* and *𝒫*) is a mapping *𝒮* FFS (*𝒵*) × FFS (*𝒵*) ⟶[0,1], possessing the following properties:

- 0 ≤*𝒮* (*𝒴* and *𝒫*) ≤ 1;
- *𝒮* (*𝒴* and *𝒫*) = *𝒮* (*𝒫* and *𝒴*);
- *𝒮* (*𝒴* and *𝒫*) = 1 iff *𝒴* = *𝒫*;
- *𝒮* (*𝒴* and *𝒴*^*c*^) = 0 iff *𝒴* is a crisp set;
- If *𝒴*⊆*𝒫*⊆*ℳ*, then *𝒮* (*𝒴* and *𝒫*) ≤*𝒮* (*𝒴* and *ℳ*) and *𝒮* (*𝒫* and *ℳ*) ≤*𝒮* (*𝒴* and *ℳ*).

**Theorem 2 .**

Let *𝒴* and *𝒫* be two FFSs, then *𝒮*_*i*_(*𝒴*, *𝒫*)(*i*=1,2,…, 13) is a distance measure.

1. ${𝒮}_{1}\left(𝒴,𝒫\right)=1-\left(1/2\left|𝒵\right|\right)\sum_{\varkappa_{i}\in 𝒵}\left(\begin{array}{c} \left|{ℑ}_{𝒴}^{3}\left(\varkappa_{i}\right)-{ℑ}_{𝒫}^{3}\left(\varkappa_{i}\right)\right|\\ +\left|{ð}_{𝒴}^{3}\left(\varkappa_{i}\right)-{ð}_{𝒫}^{3}\left(\varkappa_{i}\right)\right|+\left|\pi_{𝒴}^{3}\left(\varkappa_{i}\right)-\pi_{𝒫}^{3}\left(\varkappa_{i}\right)\right| \end{array}\right)$
2. *𝒮*_2_(*𝒴*, *𝒫*)=1 − (1/2|*𝒵*|)∑_ϰ_*i*_∈*𝒵*_(|*ℑ*_*𝒴*_^3^(ϰ_*i*_) − *ℑ*_*𝒫*_^3^(ϰ_*i*_) − (ð_*𝒴*_^3^(ϰ_*i*_) − ð_*𝒫*_^3^(ϰ_*i*_))|);
3. *𝒮*_3_(*𝒴*, *𝒫*)=1 − (1/4|*X*|)(∑_ϰ_*i*_∈*𝒵*_(|*ℑ*_*𝒴*_^3^(ϰ_*i*_) − *ℑ*_*𝒫*_^3^(ϰ_*i*_)|+|ð_*𝒴*_^3^(ϰ_*i*_) − ð_*𝒫*_^3^(ϰ_*i*_)|+|*π*_*𝒴*_^3^(ϰ_*i*_) − *π*_*𝒫*_^3^(ϰ_*i*_)|)+∑_ϰ_*i*_∈*𝒵*_(|(*ℑ*_*𝒴*_^3^(ϰ_*i*_) − ð_*𝒴*_^3^(ϰ_*i*_))+(*ℑ*_*𝒫*_^3^(ϰ_*i*_) − ð_*𝒫*_^3^(ϰ_*i*_))|))
4. *𝒮*_4_(*𝒴*, *𝒫*)=1 − (1/|*𝒵*|)∑_ϰ_*i*_∈*𝒵*_(|*ℑ*_*𝒴*_^3^(ϰ_*i*_) − *ℑ*_*𝒫*_^3^(ϰ_*i*_)|∨|(ð_*𝒴*_^3^(ϰ_*i*_) − ð_*𝒫*_^3^(ϰ_*i*_))|);
5. *𝒮*_5_(*𝒴*, *𝒫*)=(1/|*𝒵*|)∑_ϰ_*i*_∈*𝒵*_(1 − |*ℑ*_*𝒴*_^3^(ϰ_*i*_) − *ℑ*_*𝒫*_^3^(ϰ_*i*_)|∨|ð_*𝒴*_^3^(ϰ_*i*_) − ð_*𝒫*_^3^(ϰ_*i*_)|)/(1+|*ℑ*_*𝒴*_^3^(ϰ_*i*_) − *ℑ*_*𝒫*_^3^(ϰ_*i*_)|∨|ð_*𝒴*_^3^(ϰ_*i*_) − ð_*𝒫*_^3^(ϰ_*i*_)|);
6. *𝒮*_6_(*𝒴*, *𝒫*)=(∑_ϰ_*i*_∈*𝒵*_(1 − |*ℑ*_*𝒴*_^3^(ϰ_*i*_) − *ℑ*_*𝒫*_^3^(ϰ_*i*_)|∨|ð_*𝒴*_^3^(ϰ_*i*_) − ð_*𝒫*_^3^(ϰ_*i*_)|))/(∑_ϰ_*i*_∈*𝒵*_(1+|*ℑ*_*𝒴*_^3^(ϰ_*i*_) − *ℑ*_*𝒫*_^3^(ϰ_*i*_)|∨|ð_*𝒴*_^3^(ϰ_*i*_) − ð_*𝒫*_^3^(ϰ_*i*_)|));
7. *𝒮*_7_(*𝒴*, *𝒫*)=*α*(∑_ϰ_*i*_∈*𝒵*_(*ℑ*_*𝒴*_^3^(ϰ_*i*_)∧*ℑ*_*𝒫*_^3^(ϰ_*i*_))/∑_ϰ_*i*_∈*𝒵*_(*ℑ*_*𝒴*_^3^(ϰ_*i*_)∨*ℑ*_*𝒫*_^3^(ϰ_*i*_)))+*β*(∑_ϰ_*i*_∈*𝒵*_(ð_*𝒴*_^3^(ϰ_*i*_)∧ð_*𝒫*_^3^(ϰ_*i*_))/∑_ϰ_*i*_∈*𝒵*_(ð_*𝒴*_^3^(ϰ_*i*_)∨ð_*𝒫*_^3^(ϰ_*i*_))), *α*+*β*=1, *α*, *β* ∈ [0,1];
8. *𝒮*_8_(*𝒴*, *𝒫*)=(*α*/|*𝒵*|)∑_ϰ_*i*_∈*𝒵*_((*ℑ*_*𝒴*_^3^(ϰ_*i*_)∧*ℑ*_*𝒫*_^3^(ϰ_*i*_))/(*ℑ*_*𝒴*_^3^(ϰ_*i*_)∨*ℑ*_*𝒫*_^3^(ϰ_*i*_)))+(*β*/|*𝒵*|)∑_ϰ_*i*_∈*𝒵*_((ð_*𝒴*_^3^(ϰ_*i*_)∧ð_*𝒫*_^3^(ϰ_*i*_))/(ð_*𝒴*_^3^(ϰ_*i*_)∨ð_*𝒫*_^3^(ϰ_*i*_))), *α*+*β*=1, *α*, *β* ∈ [0,1];
9. *𝒮*_9_(*𝒴*, *𝒫*)=(1/|*𝒵*|)∑_ϰ_*i*_∈*𝒵*_((*ℑ*_*𝒴*_^3^(ϰ_*i*_)∧*ℑ*_*𝒫*_^3^(ϰ_*i*_))+(ð_*𝒴*_^3^(ϰ_*i*_)∧ð_*𝒫*_^3^(ϰ_*i*_)))/((*ℑ*_*𝒴*_^3^(ϰ_*i*_)∨*ℑ*_*𝒫*_^3^(ϰ_*i*_))+(ð_*𝒴*_^3^(ϰ_*i*_)∨ð_*𝒫*_^3^(ϰ_*i*_)));
10. *𝒮*_10_(*𝒴*, *𝒫*)=(∑_ϰ_*i*_∈*𝒵*_(*ℑ*_*𝒴*_^3^(ϰ_*i*_)∧*ℑ*_*𝒫*_^3^(ϰ_*i*_))+(ð_*𝒴*_^3^(ϰ_*i*_)∧ð_*𝒫*_^3^(ϰ_*i*_)))/(∑_ϰ_*i*_∈*𝒵*_(*ℑ*_*𝒴*_^3^(ϰ_*i*_)∨*ℑ*_*𝒫*_^3^(ϰ_*i*_))+(ð_*𝒴*_^3^(ϰ_*i*_)∨ð_*𝒫*_^3^(ϰ_*i*_)));
11. *𝒮*_11_(*𝒴*, *𝒫*)=(1/|*𝒵*|)∑_ϰ_*i*_∈*𝒵*_((*ℑ*_*𝒴*_^3^(ϰ_*i*_)∧*ℑ*_*𝒫*_^3^(ϰ_*i*_))+((1 − ð_*𝒴*_^3^(ϰ_*i*_))∧(1 − ð_*𝒫*_^3^(ϰ_*i*_))))/((*ℑ*_*𝒴*_^3^(ϰ_*i*_)∨*ℑ*_*𝒫*_^3^(ϰ_*i*_))+((1 − ð_*𝒴*_^3^(ϰ_*i*_))∨(1 − ð_*𝒫*_^3^(ϰ_*i*_))));
12. *𝒮*_12_(*𝒴*, *𝒫*)=(∑_ϰ_*i*_∈*𝒵*_(*ℑ*_*𝒴*_^3^(ϰ_*i*_)∧*ℑ*_*𝒫*_^3^(ϰ_*i*_))+((1 − ð_*𝒴*_^3^(ϰ_*i*_))∧(1 − ð_*𝒫*_^3^(ϰ_*i*_))))/(∑_ϰ_*i*_∈*𝒵*_(*ℑ*_*𝒴*_^3^(ϰ_*i*_)∨*ℑ*_*𝒫*_^3^(ϰ_*i*_))+((1 − ð_*𝒴*_^3^(ϰ_*i*_))∨(1 − ð_*𝒫*_^3^(ϰ_*i*_))));
13. ${𝒮}_{13}\left(𝒴,𝒫\right)=\sqrt[{t}]{\begin{array}{c} \left(1/2\left|𝒵\right|{\left(l_{1}+1\right)}^{t}\right)\sum_{\varkappa_{i}\in 𝒵}\left\{{\left|\left(l_{1}\left({ℑ}_{𝒴}^{3}\left(\varkappa_{i}\right)\right)-{ℑ}_{𝒫}^{3}\left(\varkappa_{i}\right)\right)-\left({ð}_{𝒴}^{3}\left(\varkappa_{i}\right)-{ð}_{𝒫}^{3}\left(\varkappa_{i}\right)\right)\right|}^{t}\right\}\\ +\left(1/2\left|𝒵\right|\left(l_{2}+1\right)t\right)\sum_{\varkappa_{i}\in 𝒵}\left\{{\left|\left(l_{2}\left({ð}_{𝒴}^{3}\left(\varkappa_{i}\right)\right)-{ð}_{𝒫}^{3}\left(\varkappa_{i}\right)\right)-\left({ℑ}_{𝒴}^{3}\left(\varkappa_{i}\right)-{ℑ}_{𝒫}^{3}\left(\varkappa_{i}\right)\right)\right|}^{t}\right\} \end{array}}$

**Theorem 3 .**

For *i*=1,2,3,…, 13, if *α*=*β*=1/2, then we have

- *𝒮*_*i*_(*𝒴*, *𝒫*^*c*^)=*𝒮*_*i*_(*𝒫*^*c*^, *𝒴*);
- *𝒮*_*i*_(*𝒴*, *𝒫*)=*𝒮*_*i*_(*𝒴*∩*𝒫*, *𝒴* ∪ *𝒫*);
- *𝒮*_*i*_(*𝒴*, *𝒴*∩*𝒫*)=*𝒮*_*i*_(*𝒴*, *𝒴* ∪ *𝒫*);
- *𝒮*_*i*_(*𝒴*, *𝒴* ∪ *𝒫*)=*𝒮*_*i*_(*𝒴*, *𝒴*∩*𝒫*).

**Theorem 4 .**

For *i*=1,2,…, 6, we have

- *𝒮*_*i*_(*𝒴*, *𝒴* ⊗ *𝒫*)=*𝒮*_*i*_(*𝒫*, *𝒴* ⊕ *𝒫*)*;*
- *𝒮*_*i*_(*𝒴*, *𝒴* ⊕ *𝒫*)=*𝒮*_*i*_(*𝒫*, *𝒴* ⊗ *𝒫*)*;*

**Theorem 5 .**

For *i*=1,4,5,6 and for all ϰ_*i*_ ∈ *𝒵*, (*ℑ*_*𝒴*_^3^(ϰ_*i*_)+*ℑ*_*𝒫*_^3^(ϰ_*i*_))=1, and ð_*𝒴*_^3^(ϰ_*i*_)+ð_*𝒫*_^3^(ϰ_*i*_)=1, we have

- *𝒮*_*i*_(*𝒴*, *𝒴*⊘*𝒫*)=*𝒮*_*i*_(*𝒫*, *𝒴* ⊖ *𝒫*),*ℑ*_*𝒴*_^3^(ϰ_*i*_) ≤ *ℑ*_*𝒫*_^3^(ϰ_*i*_), *and*(ð_*𝒴*_^3^(ϰ_*i*_) ≥ ð_*𝒫*_^3^(ϰ_*i*_))
- *𝒮*_*i*_(*𝒴*, *𝒴* ⊖ *𝒫*)=*𝒮*_*i*_(*𝒫*, *𝒴*⊘*𝒫*),*ℑ*_*𝒴*_^3^(ϰ_*i*_) ≥ *ℑ*_*𝒫*_^3^(ϰ_*i*_), *and*(ð_*𝒴*_^3^(ϰ_*i*_) ≤ ð_*𝒫*_^3^(ϰ_*i*_))

### 3.3. Entropy for FFSs

Let *𝒴* and *𝒫* two FFSs on *𝒵*. An entropy measure *E*(*𝒴*) is a mapping *E*: FFS (*𝒵*)⟶[0,1], carrying the following features:

(i) 0 ≤ *E*(*𝒴*) ≤ 1;
(ii) *E*(*𝒴*)=0 iff *𝒴* is a crisp set;
(iii) *E*(*𝒴*)=1 iff *ℑ*_*𝒴*_^3^(ϰ_*i*_)=ð_*𝒴*_^*t*^(ϰ_*i*_);
(iv) *E*(*𝒴*)=*E*(*𝒴*^*c*^);
(v) If *E*(*𝒴*) ≤ *E*(*𝒫*) if *𝒴* is less fuzzy than *𝒫*, that is
  (6) $$\begin{array}{c} I_{Y}^{3}\left(\varkappa_{i}\right)\leq I_{P}^{3}\left(\varkappa_{i}\right)\leq {ð}_{P}^{3}\left(\varkappa_{i}\right)\leq {ð}_{Y}^{3}\left(\varkappa_{i}\right)\text{,}\\ {ð}_{Y}^{3}\left(\varkappa_{i}\right)\leq {ð}_{P}^{3}\left(\varkappa_{i}\right)\leq I_{P}^{3}\left(\varkappa_{i}\right)\leq I_{Y}^{3}\left(\varkappa_{i}\right)\text{.} \end{array}$$

**Theorem 6 .**

*Let𝒴be two FFSs, thenE*_*i*_(*𝒴*, *𝒫*)(*i*=1,2,…, 12)*is an entropy.*

### 3.4. Inclusion Measure for FFSs

Let *𝒴*, *𝒫*, and *ℳ* be three FFSs on *𝒵*. An inclusion measure *ℐ*(*𝒴*, *𝒫*) is a mapping *ℐ*: FFS (*𝒵*)× FFS (*𝒵*)⟶[0,1], carrying the following features:

- 0 ≤ *ℐ*(*𝒴*, *𝒫*) ≤ 1;
- *ℐ*(*𝒴*, *𝒫*)=1 iff *𝒴*⊆*𝒫*;
- *ℐ*(*𝒴*, *𝒫*)=0 iff *𝒴*=Φ and *𝒫*=∅;
- If *𝒴*⊆*𝒫*⊆*ℳ*, then *ℐ*(*𝒴*, *𝒫*) ≤ *ℐ*(*𝒴*, *ℳ*) and *ℐ*(*𝒫*, *ℳ*) ≤ *ℐ*(*𝒴*, *ℳ*).

**Theorem 7 .**

Let *𝒴* and *𝒫* be two FFS *s*, then *ℐ*_*i*_(*𝒴*, *𝒫*)(*i*=1,2,…, 7) is an inclusion measure.

1. *ℐ*_1_(*𝒴*, *𝒫*)=1 − (1/2|*𝒵*|)∑_ϰ_*i*_∈*𝒵*_(|*ℑ*_*𝒴*_^3^(ϰ_*i*_) − *ℑ*_*𝒴*_^3^(ϰ_*i*_)∧*ℑ*_*𝒫*_^3^(ϰ_*i*_)|+|ð_*𝒴*_^3^(ϰ_*i*_) − ð_*𝒴*_^3^(ϰ_*i*_)∨ð_*𝒫*_^3^(ϰ_*i*_)|);
2. *ℐ*_2_(*𝒴*, *𝒫*)={1,*𝒴*=∅(∑_ϰ_*i*_∈*𝒵*_(1+*ℑ*_*𝒴*_^3^(ϰ_*i*_)∧*ℑ*_*𝒫*_^3^(ϰ_*i*_) − ð_*𝒴*_^3^(ϰ_*i*_)∨ð_*𝒫*_^3^(ϰ_*i*_)))/(∑_ϰ_*i*_∈*𝒵*_(1+*ℑ*_*𝒴*_^3^(ϰ_*i*_) − ð_*𝒴*_^3^(ϰ_*i*_))),*𝒴* ≠ ∅;
3. *ℐ*_3_(*𝒴*, *𝒫*)={1,*𝒴*=*𝒫*=∅(∑_ϰ_*i*_∈*𝒵*_(1+*ℑ*_*𝒴*_^3^(ϰ_*i*_) − ð_*𝒴*_^3^(ϰ_*i*_)))/(∑_ϰ_*i*_∈*𝒵*_(1+*ℑ*_*𝒴*_^3^(ϰ_*i*_)∨*ℑ*_*𝒫*_^3^(ϰ_*i*_) − ð_*𝒴*_^3^(ϰ_*i*_)∧ð_*𝒫*_^3^(ϰ_*i*_))),others;
4. *ℐ*_4_(*𝒴*, *𝒫*)={1,*𝒴*=*𝒫*=∅(∑_ϰ_*i*_∈*𝒵*_(1+*ℑ*_*𝒴*_^3^(ϰ_*i*_) − ð_*𝒴*_^3^(ϰ_*i*_)))/(∑_ϰ_*i*_∈*𝒵*_(1+ð_*𝒴*_^3^(ϰ_*i*_)∧ð_*𝒫*_^3^(ϰ_*i*_) − *ℑ*_*𝒴*_^3^(ϰ_*i*_)∨*ℑ*_*𝒫*_^3^(ϰ_*i*_))),others;
5. *ℐ*_5_(*𝒴*, *𝒫*)={1,*𝒴*=*𝒫*=∅(1/|*𝒵*|)∑_ϰ_*i*_∈*𝒵*_(1+*ℑ*_*𝒴*_^3^(ϰ_*i*_)∧*ℑ*_*𝒫*_^3^(ϰ_*i*_) − ð_*𝒴*_^3^(ϰ_*i*_)∨ð_*𝒫*_^3^(ϰ_*i*_))/(1+*ℑ*_*𝒴*_^3^(ϰ_*i*_) − ð_*𝒴*_^3^(ϰ_*i*_)),others
6. *ℐ*_6_(*𝒴*, *𝒫*)={1,*𝒴*=*𝒫*=∅(1/|*𝒵*|)∑_ϰ_*i*_∈*𝒵*_(1+*ℑ*_*𝒴*_^3^(ϰ_*i*_) − ð_*𝒴*_^3^(ϰ_*i*_))/(1+*ℑ*_*𝒴*_^3^(ϰ_*i*_)∨*ℑ*_*𝒫*_^3^(ϰ_*i*_) − ð_*𝒴*_^3^(ϰ_*i*_)∧ð_*𝒫*_^3^(ϰ_*i*_)),others
7. *ℐ*_7_(*𝒴*, *𝒫*)={1,*𝒴*=*𝒫*=∅(1/|*𝒵*|)∑_ϰ_*i*_∈*𝒵*_(1+*ℑ*_*𝒴*_^3^(ϰ_*i*_) − ð_*𝒴*_^3^(ϰ_*i*_))/(1+ð_*𝒴*_^3^(ϰ_*i*_)∧ð_*𝒫*_^3^(ϰ_*i*_) − *ℑ*_*𝒴*_^3^(ϰ_*i*_)∨*ℑ*_*𝒫*_^3^(ϰ_*i*_)),others

### 3.5. The Relations between These Measures

In this section, we study the relations between inclusion, entropy, similarity measure, and distance measure of Fermatean fuzzy sets. First, according to the definitions of similarity measure and distance measure of Fermatean fuzzy sets, one should note that they are all used for estimating the degree of similarity between two Fermatean fuzzy sets. The main difference is as follows: for the similarity measure, a greater value means that the two Fermatean fuzzy sets are more similar than are a pair with a lower value. The situation for the distance measure is just the opposite, that is, the smaller the value is, the more similar these two Fermatean fuzzy sets are. So, we can obtain the following theorem.

### 3.6. Transformation Relationships among Information Measures for FFSs

**Theorem 8 .**

Suppose *𝒟* be the Fermatean fuzzy distance measure for *𝒴*, *𝒫*∈ FFS s, then *𝒮*(*𝒴*, *𝒫*)=1 − *𝒟*(*𝒴*, *𝒫*) is the similarity measure of FFS s*𝒴* and *𝒫*. The proof is straightforward.

**Theorem 9 .**

For *𝒴*, *𝒫* ∈ *FFSs*, and we order *𝒟*_1_(*𝒴*, *𝒫*)=(1/|*𝒵*|)∑_ϰ_*i*_∈*𝒵*_(|*ℑ*_*𝒴*_^3^(ϰ_*i*_) − *ℑ*_*𝒫*_^3^(ϰ_*i*_)|+|ð_*𝒴*_^3^(ϰ_*i*_) − ð_*𝒫*_^3^(ϰ_*i*_)|+|*π*_*𝒴*_^3^(ϰ_*i*_) − *π*_*𝒫*_^3^(ϰ_*i*_)|), *then we have𝒮*(*𝒴*, *𝒫*)=1 − (1/2|*𝒵*|)∑_ϰ_*i*_∈*𝒵*_(|*ℑ*_*𝒴*_^3^(ϰ_*i*_) − *ℑ*_*𝒫*_^3^(ϰ_*i*_)|+|ð_*𝒴*_^3^(ϰ_*i*_) − ð_*𝒫*_^3^(ϰ_*i*_)|+|*π*_*𝒴*_^3^(ϰ_*i*_) − *π*_*𝒫*_^3^(ϰ_*i*_)|)=*𝒮*_1_(*𝒴*, *𝒫*).

Also, *𝒮*_*i*_(*𝒴*, *𝒫*)=1 − *𝒟*_*i*_(*𝒴*, *𝒫*)(*i*=1,2,…, 13).

**Theorem 10 .**

Let *𝒟* and *𝒮* be the distance and similarity measures of FFSs, for *𝒴*∈ FFSs, then *E*(*𝒴*)=1 − *𝒮*(*𝒴*, *𝒴*^*c*^)=1 − *𝒮*(*𝒴*, *𝒴*^*c*^) is the entropy of FFSs.

**Proof**

- (*E*_1_) It is straightforward.
- (*E*_2_) If *𝒴* is a crisp set, then *𝒴*=∅ or *𝒴*=Φ, we have *𝒮*(*𝒴*, *𝒴*^*c*^)=0. Therefore, *E*(*𝒴*)=0.
- (*E*_3_) *E*(*𝒴*)=1⇔*𝒮*(*𝒴*, *𝒴*^*c*^)=*𝒮*(*𝒴*^*c*^, *𝒴*)⇔*ℑ*_*𝒴*_^3^(ϰ_*i*_)=*ℐ*_*𝒴*_^3^(ϰ_*i*_)=ð_*𝒴*_^3^(ϰ_*i*_) for ϰ_*i*_ ∈ *𝒵*.
- (*E*_4_) *E*(*𝒴*)=*𝒮*(*𝒴*, *𝒴*^*c*^)=*𝒮*(*𝒴*^*c*^, *𝒴*)=*E*(*𝒴*^*c*^).
- (*E*_5_) Since *ℑ*_*𝒴*_^3^(ϰ_*i*_) ≤ *ℑ*_*𝒫*_^3^(ϰ_*i*_) ≤ ð_*𝒫*_^3^(ϰ_*i*_) ≤ ð_*𝒴*_^3^(ϰ_*i*_) implies *𝒴*⊆*𝒫*⊆*𝒫*^*c*^⊆*𝒴*^*c*^. Therefore, according the definition of similarity measure of FFS *s*, we have *𝒮*(*𝒴*, *𝒴*^*c*^) ≤ *𝒮*(*𝒫*, *𝒴*^*c*^) ≤ *E*(*𝒴*, *𝒴*^*c*^), that is, *E*(*𝒴*) ≤ *E*(*𝒫*). Similarly, if ð_*𝒴*_^3^(ϰ_*i*_) ≤ ð_*𝒫*_^3^(ϰ_*i*_) ≤ *ℑ*_*𝒫*_^3^(ϰ_*i*_) ≤ *ℑ*_*𝒴*_^3^(ϰ_*i*_), then we have *𝒮*(*𝒴*, *𝒴*^*c*^) ≤ *𝒮*(*𝒫*, *𝒴*^*c*^) ≤ *𝒮*(*𝒫*, *𝒫*^*c*^), that is, *E*(*𝒴*) ≤ *E*(*𝒫*). This completes the proof.

**Theorem 11 .**

For *𝒴*, *𝒫* ∈ *FFSs*, and we order

 
*𝒮*(*𝒴*, *𝒫*)=*𝒮*_1_(*𝒴*, *𝒫*)=1 − (1/2|*𝒵*|)∑_ϰ_*i*_∈*𝒵*_(|*ℑ*_*𝒴*_^3^(ϰ_*i*_) − *ℑ*_*𝒫*_^3^(ϰ_*i*_)|+|ð_*𝒴*_^3^(ϰ_*i*_) − ð_*𝒫*_^3^(ϰ_*i*_)|+|*π*_*𝒴*_^3^(ϰ_*i*_) − *π*_*𝒫*_^3^(ϰ_*i*_)|)*, then we have*
 
*E*(*𝒴*)=*𝒮*_1_(*𝒴*, *𝒴*^*c*^)=1 − (1/|*𝒵*|)∑_ϰ_*i*_∈*𝒵*_|*ℑ*_*𝒴*_^3^(ϰ_*i*_) − ð_*𝒴*_^3^(ϰ_*i*_)*|*=*E*_4_(*𝒴*)* Also,*
 
*E*_4_(*𝒴*)=*𝒮*_2_(*𝒴*, *𝒴*^*c*^)=*𝒮*_3_(*𝒴*, *𝒴*^*c*^)=*𝒮*_4_(*𝒴*, *𝒴*^*c*^)=*𝒮*_13_(*𝒴*, *𝒴*^*c*^)
 
*E*_5_(*𝒴*)=*𝒮*_7_(*𝒴*, *𝒴*^*c*^)=*𝒮*_10_(*𝒴*, *𝒴*^*c*^), *E*_6_(*𝒴*)=*𝒮*_8_(*𝒴*, *𝒴*^*c*^)=*𝒮*_9_(*𝒴*, *𝒴*^*c*^), *E*_7_(*𝒴*)=*𝒮*_12_(*𝒴*, *𝒴*^*c*^)*E*_8_(*𝒴*)=*𝒮*_11_(*𝒴*, *𝒴*^*c*^).

**Definition 10 .**

Let *𝒴* be an FFS, *m*(*𝒴*), *n*(*𝒴*)∈ FFSs, ∀ϰ_*i*_ ∈ *𝒵*, *m*(*𝒴*)(ϰ_*i*_)=(*ℑ*_*m*_(*𝒴*)(ϰ_*i*_), ð_*m*_(*𝒴*)(ϰ_*i*_)), and *n*(*𝒴*)(ϰ_*i*_)=(*ℑ*_*n*_(*𝒴*)(ϰ_*i*_), ð_*n*_(*𝒴*)(ϰ_*i*_)) their membership and nonmembership functions are defined as follows:

 
${ℑ}_{m}\left(𝒴\right)\left(\varkappa_{i}\right)=\sqrt[{3}]{1+{\left({ℑ}_{𝒴}^{3}\left(\varkappa_{i}\right)-{ð}_{𝒴}^{3}\left(\varkappa_{i}\right)\right)}^{3}}$,
 
${ð}_{m}\left(𝒴\right)\left(\varkappa_{i}\right)=\sqrt[{3}]{1-\left|{ℑ}_{𝒴}^{3}\left(\varkappa_{i}\right)-{ð}_{𝒴}^{3}\left(\varkappa_{i}\right)\right|}$,
 
${ℑ}_{n}\left(𝒴\right)\left(\varkappa_{i}\right)=\sqrt[{3}]{1-{\left({ℑ}_{𝒴}^{3}\left(\varkappa_{i}\right)-{ð}_{𝒴}^{3}\left(\varkappa_{i}\right)\right)}^{3}}$,
 
${ð}_{n}\left(𝒴\right)\left(\varkappa_{i}\right)=\sqrt[{3}]{1+\left|{ℑ}_{𝒴}^{3}\left(\varkappa_{i}\right)-{ð}_{𝒴}^{3}\left(\varkappa_{i}\right)\right|}$.

**Theorem 12 .**

Let *𝒟* be the distance measure and *𝒮* be the similarity measure of FFSs, for *𝒴* in FFSs, then *E*(*𝒴*)=*𝒮*(*m*(*𝒴*), *n*(*𝒴*))=1 − *𝒟*(*𝒴*, *𝒴*^*c*^)=1 − *𝒮*(*m*(*𝒴*), *n*(*𝒴*)) is the entropy of FFS *𝒴*.

**Proof**

(i) (*E*_1_) It is straightforward.
(ii) (*E*_2_) If *𝒴* is a crisp set, then ∀ϰ_*i*_ ∈ *𝒵*, we have *ℑ*_*𝒴*_(ϰ_*i*_)=1, ð_*𝒴*_(ϰ_*i*_)=0, or *ℑ*_*𝒴*_(ϰ_*i*_)=0, ð_*𝒴*_(ϰ_*i*_)=1. Therefore, we can achieve
  (7) $$\begin{array}{c} I_{m}\left(Y\right)\left(\varkappa_{i}\right)=1,{ð}_{m}\left(Y\right)\left(\varkappa_{i}\right)=0,I_{m}\left(Y\right)\left(\varkappa_{i}\right)=0,{ð}_{m}\left(Y\right)\left(\varkappa_{i}\right)=1\text{.} \end{array}$$
It implies that *m*(*𝒴*)=Φ, *n*(*𝒴*)=∅, consequently, *𝒮*(*m*(*𝒴*), *n*(*𝒴*))=0.
(iii) (*E*_3_) $\begin{array}{ccc} E\left(𝒴\right) & = & 1\Leftrightarrow 𝒮\left(m\left(𝒴\right),n\left(𝒴\right)\right)=1\Leftrightarrow m\left(𝒴\right)=n\left(𝒴\right)\Leftrightarrow {ℑ}_{m}\left(𝒴\right)\left(\varkappa_{i}\right)\\ & = & {ℑ}_{n}\left(𝒴\right)\left(\varkappa_{i}\right),{ð}_{m}\left(𝒴\right)\left(\varkappa_{i}\right)={ð}_{n}\left(𝒴\right)\left(\varkappa_{i}\right)\text{.} \end{array}$
(iv) (*E*_4_) Using the definitions of *m*(*𝒴*) and *n*(*𝒴*), we have *m*(*𝒴*)=*m*(*𝒴*^*c*^), *n*(*𝒴*)=*n*(*𝒴*^*c*^), hence *𝒮*(*m*(*𝒴*), *n*(*𝒴*))=*𝒮*(*m*(*𝒴*^*c*^), *n*(*𝒴*^*c*^)).
(v) (*E*_5_) Since *ℑ*_*𝒴*_(ϰ_*i*_) ≤ *ℑ*_*𝒫*_(ϰ_*i*_) ≤ ð_*𝒴*_(ϰ_*i*_) ≤ ð_*𝒫*_(ϰ_*i*_) implies *𝒴*⊆*𝒫*⊆*𝒫*^*c*^⊆*𝒴*^*c*^. Therefore, we have |*ℑ*_*𝒴*_^3^(ϰ_*i*_) − ð_*𝒴*_^3^(ϰ_*i*_)| ≥ |(*ℑ*_*𝒫*_^3^(ϰ_*i*_) − ð_*𝒫*_^3^(ϰ_*i*_))|. It means that n(A) ≤*n*(*𝒫*) ≤ *m*(*𝒫*) ≤ *m*(*𝒴*), so we have *𝒮*(*m*(*𝒴*), *n*(*𝒴*)) ≤ *𝒮*(*m*(*𝒫*), *n*(*𝒴*)) ≤ *𝒮*(*m*(*𝒫*), *n*(*𝒫*)) that is, *E*(*𝒴*) ≤ *E*(*𝒫*).

Similarly, if ð_*𝒴*_(ϰ_*i*_) ≤ ð_*𝒫*_(ϰ_*i*_) ≤ *ℑ*_*𝒫*_(ϰ_*i*_) ≤ *ℑ*_*𝒴*_(ϰ_*i*_), then we have

(8) $$\begin{array}{c} S\left(m\left(Y\right),n\left(Y\right)\right)\leq S\left(m\left(Y\right),n\left(P\right)\right)\leq S\left(m\left(P\right),n\left(P\right)\right),that\;is,E\left(Y\right)\leq E\left(P\right)\text{.} \end{array}$$

This completes the proof.

**Theorem 13 .**

Suppose *𝒟* and *𝒮* be the distance measure and similarity measures of FFSs, respectively, for *𝒴*, *𝒫*∈ FFSs, then *ℐ*(*𝒴*, *𝒫*)=*𝒮*(*𝒴*, *𝒴*∩*𝒫*)=1 − *𝒟*(*𝒴*, *𝒴*∩*𝒫*) is the inclusion measure of FFSs *𝒴* and *𝒫*.

**Proof**

- (*I*_1_) It is straightforward.
- (*I*_2_) If *𝒴*⊆*𝒫*, then *𝒮*(*𝒴*, *𝒴*∩*𝒫*)=*𝒮*(*𝒴*, *𝒴*)=1=*𝒮*(*𝒴*, *𝒫*).
- (*I*_3_) *ℐ*(*𝒴*, *𝒫*)=0⇔*𝒮*(*𝒴*, *𝒴*∩*𝒫*)=0⇔*𝒴*=Φ, *𝒫*=∅.
- (*I*_4_) If *𝒴*⊆*𝒫*⊆*𝒪*, then *ℐ*(*𝒪*, *𝒴*)=*𝒮*(*𝒪*, *𝒪*∩*𝒴*)=*𝒮*(*𝒪*, *𝒴*) and *ℐ*(*𝒫*, *𝒴*)=*𝒮*(*𝒫*, *𝒫*∩*𝒴*)=*𝒮*(*𝒫*, *𝒴*). Known by the similarity measure of FFSs, we have *ℐ*(*𝒪*, *M*) ≤ *ℐ*(*𝒫*, *𝒴*). Similarly, *ℐ*(*𝒪*, *𝒴*) ≤ *ℐ*(*𝒪*, *𝒫*). This completes the proof.

**Theorem 14 .**

Suppose *𝒟* and *𝒮* be the distance measure and similarity measures of FFSs, respectively, for *𝒴*, *𝒫* in FFSs, then *ℐ*(*𝒴*, *𝒫*)=*𝒮*(*𝒫*, *𝒴* ∪ *𝒫*)=1 − *𝒟*(*𝒫*, *𝒴* ∪ *𝒫*) is the inclusion measure of FFSs *𝒴* and *𝒫*.

**Definition 11 .**

Let *𝒴* and *𝒫* be two FFS *s*, then we define g(A,B) ∈ FFS *s*, ∀ϰ_*i*_ ∈ *𝒵*,

 
${ℑ}_{g}\left(𝒴,𝒫\right)\left(\varkappa_{i}\right)=\sqrt{\left(1+\min\;\left\{\left|{ℑ}_{𝒴}^{3}\left(\varkappa_{i}\right)-{ℑ}_{𝒫}^{3}\left(\varkappa_{i}\right)\right|,\left|{ð}_{𝒴}^{3}\left(\varkappa_{i}\right)-{ð}_{𝒫}^{3}\left(\varkappa_{i}\right)\right|\right\}/2\right)},$
 
${ð}_{g}\left(𝒴,𝒫\right)\left(\varkappa_{i}\right)=\sqrt{\left(1-\max\;\left\{\left|{ℑ}_{𝒴}^{3}\left(\varkappa_{i}\right)-{ℑ}_{𝒫}^{3}\left(\varkappa_{i}\right)\right|,\left|{ð}_{𝒴}^{3}\left(\varkappa_{i}\right)-{ð}_{𝒫}^{3}\left(\varkappa_{i}\right)\right|\right\}/2\right)}$.

**Theorem 15 .**

Suppose *E* be the entropy measure of FFSs, for *𝒴*, *𝒫*∈ FFSs, then *E*(*g*(*𝒴*, *𝒫*)) is the similarity measure of FFSs *𝒴* and *𝒫*.

**Proof**

- (*S*_1_)− ( *S*_2_) are straightforward.
- (*S*_3_) Known by the definition of entropy of FFS *s*, *E*(*g*(*𝒴*, *𝒫*))=1⇔*ℑ*_*g*_(*𝒴*, *𝒫*)(ϰ_*i*_)=ð_*g*_(*𝒴*, *𝒫*)(ϰ_*i*_)⇔|*ℑ*_*𝒴*_^3^(ϰ_*i*_) − *ℑ*_*𝒫*_^*t*^(ϰ_*i*_)|=0, |ð_*𝒴*_^3^(ϰ_*i*_) − ð_*𝒫*_^3^(ϰ_*i*_)|=0⇔*ℑ*_*𝒴*_(ϰ_*i*_)=*ℑ*_*𝒫*_(ϰ_*i*_), ð_*𝒴*_(ϰ_*i*_)=ð_*𝒫*_(ϰ_*i*_)⇔*𝒴*=*𝒫*.
- (*S*_4_) If *𝒴* is a crisp set, then *ℑ*_*𝒴*_(ϰ_*i*_)=1, ð_*𝒴*_(ϰ_*i*_)=0 or *ℑ*_*𝒴*_(ϰ_*i*_)=0, ð_*𝒴*_(ϰ_*i*_)=1. Hence, *ℑ*_*g*_(*𝒴*, *𝒴*^*c*^)(ϰ_*i*_)=1, *ℐ*_*𝒴*_(*𝒴*, *𝒫*^*c*^)(ϰ_*i*_)=0, ð_*𝒴*_(*𝒴*, *𝒫*^*c*^)(ϰ_*i*_)=0, it implies *g*(*𝒴*, *𝒫*^*c*^) = Φ, so *E*(*g*(*𝒴*, *𝒫*^*c*^))=0.
- (*S*_5_) Since *𝒴*⊆*𝒫*⊆*𝒪*, then ∀ϰ_*i*_ ∈ *𝒵*, we have *ℑ*_*𝒴*_(ϰ_*i*_) ≤ *ℑ*_*𝒫*_(ϰ_*i*_) ≤ *ℑ*_*𝒪*_(ϰ_*i*_), ð_*𝒪*_(ϰ_*i*_) ≤ ð_*𝒫*_(ϰ_*i*_) ≤ ð_*𝒴*_(ϰ_*i*_). Therefore, we have *ℑ*_*𝒴*_^3^(ϰ_*i*_) − *ℑ*_*𝒪*_^3^(ϰ_*i*_)*|* ≥ |*ℑ*_*𝒴*_^3^(ϰ_*i*_) − *ℑ*_*𝒫*_^3^(ϰ_*i*_)| and |ð_*𝒴*_^3^(ϰ_*i*_) − ð_*𝒪*_^3^(ϰ_*i*_)| ≥ |ð_*𝒴*_^3^(ϰ_*i*_) − ð_*𝒫*_^3^(ϰ_*i*_)|.

Furthermore, we have

(9) $$\begin{array}{c} \min\left\{\left|I_{Y}^{3}\left(\varkappa_{i}\right)-I_{O}^{3}\left(\varkappa_{i}\right)\right|,\left|{ð}_{Y}^{3}\left(\varkappa_{i}\right)-{ð}_{O}^{3}\left(\varkappa_{i}\right)\right|\right\},\\ \geq \min\left\{\left|I_{Y}^{3}\left(\varkappa_{i}\right)-I_{P}^{3}\left(\varkappa_{i}\right)\right|,\left|{ð}_{Y}^{3}\left(\varkappa_{i}\right)-{ð}_{P}^{3}\left(\varkappa_{i}\right)\right|\right\},\\ \max\left\{\left|I_{Y}^{3}\left(\varkappa_{i}\right)-I_{O}^{3}\left(\varkappa_{i}\right)\right|,\left|{ð}_{Y}^{3}\left(\varkappa_{i}\right)-{ð}_{O}^{3}\left(\varkappa_{i}\right)\right|\right\},\\ \geq \max\left\{\left|I_{Y}^{3}\left(\varkappa_{i}\right)-I_{P}^{3}\left(\varkappa_{i}\right)\right|,\left|{ð}_{Y}^{3}\left(\varkappa_{i}\right)-{ð}_{P}^{3}\left(\varkappa_{i}\right)\right|\right\}. \end{array}$$

Also, we can know

(10) $$\begin{array}{c} I_{g}\left(Y,O\right)\left(\varkappa_{i}\right)\geq I_{g}\left(Y,P\right)\left(\varkappa_{i}\right)\text{,}\\ {ð}_{Y}\left(Y,O\right)\left(\varkappa_{i}\right)\leq {ð}_{Y}\left(Y,P\right)\left(\varkappa_{i}\right),I_{g}\left(Y,P\right)\left(\varkappa_{i}\right)\geq {ð}_{Y}\left(Y,P\right)\left(\varkappa_{i}\right)\text{,} \end{array}$$

that is

(11) $$\begin{array}{c} {ð}_{g}\left(Y,O\right)\left(\varkappa_{i}\right)\leq {ð}_{g}\left(Y,P\right)\left(\varkappa_{i}\right)\leq I_{g}\left(Y,P\right)\left(\varkappa_{i}\right)\leq I_{g}\left(Y,O\right)\left(\varkappa_{i}\right)\text{,} \end{array}$$

and known by the definition, *E*(*g*(*𝒴*, *𝒪*)) ≤ *E*(*g*(*𝒴*, *𝒫*)).

Similarly, we can prove that *E*(*g*(*𝒴*, *𝒪*)) ≤ *E*(*g*(*𝒫*, *𝒪*)). This completes the proof. □

**Theorem 16 .**

Suppose *ℐ* be the inclusion measure of FFS *s*, for *𝒴*∈ FFS *s*, then *E*(*𝒴*)=*ℐ*(*𝒴* ∪ *𝒴*^*c*^, *𝒴*∩*𝒴*^*c*^) is the entropy of FFS *s* *𝒴*.

**Proof**

- (*E*_1_) It is straightforward.
- (*E*_2_) If *𝒴* is a crisp set, then *𝒴*=Φ or *𝒴*=∅, we have *ℐ*(*𝒴* ∪ *𝒴*^*c*^, *𝒴*∩*𝒴*^*c*^)=*ℐ*(Φ, ∅)=0. Therefore, *E*(*𝒴*)=0.
- (*E*_3_) *E*(*𝒴*) = 1 ⇔*ℐ*(*𝒴* ∪ *𝒴*^*c*^, *𝒴*∩*𝒴*^*c*^)=1⇔*𝒴* ∪ *𝒴*^*c*^⊆*𝒴*∩*𝒴*^*c*^⇔*𝒴* ∪ *𝒴*^*c*^=*𝒴*∩*𝒴*^*c*^⇔*ℑ*_*𝒴*_(ϰ_*i*_)=*ℐ*_*𝒴*_(ϰ_*i*_)=ð_*𝒴*_(ϰ_*i*_)
- (*E*_4_) *E*(*𝒴*)=*ℐ*(*𝒴* ∪ *𝒴*^*c*^, *𝒴*∩*𝒴*^*c*^)=*ℐ*(*𝒴*^*c*^ ∪ *𝒴*, *𝒴*^*c*^∩*𝒴*)=*E*(*𝒴*^*c*^).
- (*E*_5_) Since *ℑ*_*𝒴*_(ϰ_*i*_) ≤ *ℑ*_*𝒫*_(ϰ_*i*_) ≤ *ℐ*_*𝒫*_(ϰ_*i*_) ≤ *ℐ*_*𝒴*_(ϰ_*i*_) ≤ ð_*𝒫*_(ϰ_*i*_) ≤ ð_*𝒴*_(ϰ_*i*_) implies *𝒴*⊆*𝒫*⊆*𝒫*^*c*^⊆*𝒴*^*c*^. Furthermore, *𝒴*∩*𝒴*^*c*^⊆*𝒫*∩*𝒫*^*c*^⊆*𝒫* ∪ *𝒫*^*c*^⊆*𝒴* ∪ *𝒴*^*c*^.

According to the definition of inclusion measure, we have *ℐ*(*𝒴* ∪ *𝒴*^*c*^, *𝒴*∩*𝒴*^*c*^) ≤ *ℐ*(*𝒴* ∪ *𝒴*^*c*^, *𝒫*∩*𝒫*^*c*^) ≤ *ℐ*(*𝒫* ∪ *𝒫*^*c*^, *𝒫*∩*𝒫*^*c*^), so *E*(*𝒴*) ≤ *E*(*𝒫*).

Similarly, if ð_*𝒴*_(ϰ_*i*_) ≤ ð_*𝒫*_(ϰ_*i*_) ≤ *ℑ*_*𝒫*_(ϰ_*i*_) ≤ *ℑ*_*𝒴*_(ϰ_*i*_), then we can have *ℐ*(*𝒴* ∪ *𝒴*^*c*^, *𝒴*∩*𝒴*^*c*^) ≤ *ℐ*(*𝒫* ∪ *𝒫*^*c*^, *𝒴*∩*𝒴*^*c*^) ≤ *ℐ*(*𝒫* ∪ *𝒫*^*c*^, *𝒫*∩*𝒫*^*c*^), that is E(A) ≤*E*(*𝒫*). This completes the proof.

**Example 1 .**

For *𝒴*, *𝒫*∈ FFS *s*, and we order *ℐ*(*𝒴*, *𝒫*)=*ℐ*_1_(*𝒴*, *𝒫*)=1 − (1/2|*𝒵*|)∑_ϰ_*i*_∈*𝒵*_(|*ℑ*_*𝒴*_^3^(ϰ_*i*_) − *ℑ*_*𝒴*_^3^(ϰ_*i*_)∧*ℑ*_*𝒫*_^3^(ϰ_*i*_)|+|ð_*𝒴*_^3^(ϰ_*i*_) − ð_*𝒴*_^3^(ϰ_*i*_)|), then we have *E*(*𝒴*)=*ℐ*_1_(*𝒴* ∪ *𝒴*^*c*^, *𝒴*∩*𝒴*^*c*^)=1 − (1/2|*𝒵*|)∑_ϰ_*i*_∈*𝒵*_(|*ℑ*_*𝒴*_^3^(ϰ_*i*_) − ð_*𝒴*_^3^(ϰ_*i*_)|)=*E*_4_(*𝒴*)

**Theorem 17 .**

*𝒮*(*ℑ*_*𝒴*_(ϰ_*i*_), ð_*𝒴*_(ϰ_*i*_))*is the entropy of FFS𝒴*.

**Proof**

(i) (*E*_1_) It is straightforward.
(ii) (*E*_2_) If *𝒴* is a crisp set, then we have *ℑ*_*𝒴*_(ϰ_*i*_)=Φ, and ð_*𝒴*_(ϰ_*i*_)=∅ or *ℑ*_*𝒴*_(ϰ_*i*_)=∅, and ð_*𝒴*_(ϰ_*i*_)=Φ. Therefore, *𝒮*(*ℑ*_*𝒴*_(ϰ_*i*_), ð_*𝒴*_(ϰ_*i*_)=0.
(iii) (*E*_3_) Known by the definition of similarity measure of FFSs, we have
  (12) $$\begin{array}{c} S\left(I_{Y}\left(\varkappa_{i}\right),{ð}_{Y}\left(\varkappa_{i}\right)\right)=1\Leftrightarrow I_{Y}\left(\varkappa_{i}\right)={ð}_{Y}\left(\varkappa_{i}\right)\Leftrightarrow E\left(Y\right)=1\text{.} \end{array}$$
(iv) (*E*_4_) *E*(*𝒴*)=*𝒮*(*ℑ*_*𝒴*_(ϰ_*i*_), ð_*𝒴*_(ϰ_*i*_))=*𝒮*(ð_*𝒴*_(ϰ_*i*_), *ℑ*_*𝒴*_(ϰ_*i*_))=*E*(*𝒴*^*c*^).
(v) (*E*_5_) Since *ℑ*_*𝒴*_(ϰ_*i*_) ≤ *ℑ*_*𝒫*_(ϰ_*i*_) ≤ ð_*𝒫*_(ϰ_*i*_) ≤ ð_*𝒴*_(ϰ_*i*_) implies *𝒴*⊆*𝒫*⊆*𝒫*^*c*^⊆*𝒴*^*c*^. Namely, *ℑ*_*𝒴*_⊆*ℑ*_*𝒫*_⊆ð_*𝒫*_⊆ð_*𝒴*_. According to the definition of similarity measure, we can have *𝒮*(*ℑ*_*𝒴*_(ϰ_*i*_), ð_*𝒴*_(ϰ_*i*_)) ≤ *𝒮*(*ℑ*_*𝒫*_(ϰ_*i*_), ð_*𝒫*_(ϰ_*i*_)), that is, *E*(*𝒴*) ≤ *E*(*𝒫*).

Similarly, if ð_*𝒴*_(ϰ_*i*_) ≤ ð_*𝒫*_(ϰ_*i*_) ≤ *ℑ*_*𝒫*_(ϰ_*i*_) ≤ *ℑ*_*𝒴*_(ϰ_*i*_), then we can have *E*(*𝒴*) ≤ *E*(*𝒫*). This completes the proof.

## 4. Apply the Distance Measure between FFSs to Pattern Recognition

In this part, we use numerical examples to show the feasibility and effectiveness of the innovative FFS distance measures to illustrate the applications of the established distance measures for specific FFSs in pattern recognition. Furthermore, we compare them to the existing distance measures.

**Example 2 .**

Let *ℳ*_1_, *ℳ*_2_, *ℳ*_3_, and *ℳ*_4_ be four known patterns that are illustrated by the following FFSs in *X* as follows:

(13) $$\begin{array}{c} M_{1}=\left\{\left(\varkappa_{1},0.32,0.36\right),\left(\varkappa_{2},0.41,0.47\right),\left(\varkappa_{3},0.54,0.48\right)\right\}\text{,}\\ M_{2}=\left\{\left(\varkappa_{1},0.41,0.43\right),\left(\varkappa_{2},0.52,0.51\right),\left(\varkappa_{3},0.60,0.32\right)\right\}\text{,}\\ M_{3}=\left\{\left(\varkappa_{1},0.34,0.57\right),\left(\varkappa_{2},0.56,0.61\right),\left(\varkappa_{3},0.86,0.81\right)\right\}\text{,}\\ M_{4}=\left\{\left(\varkappa_{1},0.23,0.11\right),\left(\varkappa_{2},0.31,0.30\right),\left(\varkappa_{3},0.45,0.35\right)\right\}\text{,}\\ \begin{array}{c} L=\left\{\left(\varkappa_{1},0.25,0.22\right),\left(\varkappa_{2},0.46,0.34\right),\left(\varkappa_{3},0.47,0.54\right)\right\}, \end{array} \end{array}$$

where *ℒ* is an unknown pattern. Its aimed is to determine the class to which *ℒ* belongs. In order to do that, the distance between *ℒ* and classes *ℳ*_1_, *ℳ*_2_, *ℳ*_3_, and *ℳ*_4_ are measured, and *ℒ* is then allocated to the class *ℳ*_*g*_ specified as follows:

(14) $$\begin{array}{c} g=\underset{g}{\mathrm{argmax}}\left\{D\left(M_{g},L\right)\right\}\text{.} \end{array}$$

For all the newly developed distance measures (*𝒟*_1_ − *𝒟*_13_) for FFS, the distance between *𝒟*(*ℳ*_1_, *ℒ*), *𝒟*(*ℳ*_2_, *ℒ*), *𝒟*(*ℳ*_3_, *ℒ*), and *𝒟*(*ℳ*_4_, *ℒ*) is determined and displayed in Table 1. It is observed in the Table 1, that an unknown pattern *ℒ* belongs to a class *ℳ*_3_ when *𝒟*_1_ to *𝒟*_13_ are used. It is clear that the cause for this difference is the first characteristic, i.e., (ϰ_1_). The FFNs of ϰ_1_ are as follows:

(15) $$\begin{array}{c} \begin{array}{ccc} \left(0.32,0.36\right)\text{,} & \left(0.41,0.43\right)\text{,} & \left(0.34,0.57\right)\text{,}\\ \left(0.23,0.11\right)\text{,} & \left(0.25,0.22\right)\text{,} & \; \end{array} \end{array}$$

for *ℳ*_1_, *ℳ*_2_, *ℳ*_3_, *ℳ*_4_, and *ℒ*, respectively. It is predicted that the distance between (0.34,0.57) and (0.25,0.22) is larger than the distance between (0.41,0.43) and (0.25,0.22) is larger than the distance between (0.23,0.11) and (0.25,0.22) is larger than (0.32,0.36) and (0.25,0.22). As a conclusion, it appears that

(16) $$\begin{array}{c} D\left(M_{3},L\right)>D\left(M_{2},L\right)>D\left(M_{4},L\right)>D\left(M_{4},L\right)\text{,} \end{array}$$

is more acceptable. By routine calculations, we can find the aforementioned relation for *𝒟* to *𝒟*_13_ as shown in Table 1.

**Example 3 .**

Assume that a doctor would like to diagnose the condition of *C*:  (viral fever, malaria, typhoid, or chest problem) for patients *P*:  (Ragu, Mathi,Velu, and Karthi) with disease symptoms V: (headache, acidity, burning eyes and depression). The symptoms associated with the considered diagnosis are listed in Table 2–4, and the symptoms of the disease associated with each patient are listed in Table 2. Each table element is represented by a specific FFSs. For each patient, a precise diagnosis is necessary.The distance measuring methods mentioned here are used to assess the distance between each patient and each diagnosis. Each patient was then diagnosed using the concept of the shortest possible distance. To determine a condition of the patient, we may assess the distance measure between the symptoms associated with each illness and those associated with the patient. The diagnostic findings are provided in Table 2–4 using the distance measure formula *𝒟*_13_. We may conclude that all the patients suffer from viral fever.

### 4.1. Comparison of the Distance Measure between FFSs in Medical Diagnosis

To illustrate the effectiveness of the novel distance measure for specific FFSs in pattern recognition, we present a numerical example and compare the novel findings to those reported in the literature.

**Example 4 .**

Consider a set of four patients, Al, Bob, Joe, and Ted, as represented by *ℳ*={*ℳ*_1_, *ℳ*_2_, *ℳ*_3_, *ℳ*_4_} who have the symptoms temperature, headache, stomach pain, cough, and chest pain, which are represented by *S*={ϰ_1_, ϰ_2_, ϰ_3_, ϰ_4_, ϰ_5_}. Let *ℒ*={*ℒ*_1_, *ℒ*_2_, *ℒ*_3_, *ℒ*_4_, *ℒ*_5_} be a list of possible diagnoses. Possible disease are defined as follows: *ℒ*_1_: viral fever, *ℒ*_2_: malaria, *ℒ*_3_: typhoid, *ℒ*_4_: stomach problem, and *ℒ*_5_: chest problem. The FF relation *ℳ*⟶*S* is illustrated by FFS, as can be seen in Table 5. The FF relation *S* ⟶*ℒ* is denoted by the FFS, as seen in Table 6. Each element in Table 6 is represented by FFS. The established distance measure methods are used to determine the distance between each patient and each diagnosis. Then, using the idea of minimal distance degree, each patient was diagnosed. We demonstrated the distance measure results of the patient *ℳ*_*j*_(*j*=1,2,3,4) with regard to the diagnostic *ℒ*_*i*_(*i*=1,2,3,4,5) and the final diagnosis findings are given in Al has malaria Table 7, Bob has stomach problem Table 8, Joe has typhoid Table 9, and Ted has viral fever Table 10. We perform a comparison study with other methodologies to demonstrate the capability and validity of the presented distance measures, and the findings are provided in Table 11. Table 11 shows that the suggested distance measure approaches achieve the same result as in *𝒟* [18], *𝒟* [35], *𝒟* [36], and *𝒟* [37], demonstrating that using the proposed distance measure methods to solve the medical diagnosis problem is possible and beneficial. From the preceding practical implementation of the measures techniques, we may deduce that the proposed distance measures approaches are more effective and superior in handling real world challenges.

## 5. Apply the Similarity Measure between FFSs to Pattern Recognition

In this part, we describe some examples to show the use of the suggested similarity measures based on FFS to pattern recognition.

**Example 5 .**

Suppose the four classes *ℳ*_1_, *ℳ*_2_, *ℳ*_3_, and *ℳ*_4_ of known construction materials and *ℒ*, an unknown construction material, are defined in the space *X*={ϰ_1_, ϰ_2_, ϰ_3_} and are represented by FFS is given. Its goal is to ascertain to which class *ℒ* belongs to (see Table 12).

Here, *ℒ* is a known building materials. Its objective is to determine the class to which *ℒ* belongs. To do this, the degrees of similarity between *ℒ* and classes *ℳ*_1_, *ℳ*_2_, *ℳ*_3_, and *ℳ*_4_ are measured, and *ℒ* is then allocated to the class *ℳ*_*g*_ specified as follows:

(17) $$\begin{array}{c} g=\underset{g}{\mathrm{argmax}}\left\{S\left(M_{g},L\right)\right\}\text{.} \end{array}$$

For all the established similarity measure (*𝒮*_1_ − *𝒮*_13_) for FFS, the degree of similarity between the four classes of known building materials *𝒮*(*ℳ*_1_, *ℒ*), *𝒮*(*ℳ*_2_, *ℒ*), *𝒮*(*ℳ*_3_, *ℒ*), and *𝒮*(*ℳ*_4_, *ℒ*) are determined and displayed in Table 13. It is clearly observed in the Table 13, that an unknown building material *ℒ* belongs to a class *ℳ*_1_ when *𝒮*_1_, *𝒮*_3_, *𝒮*_7_, to *𝒮*_10_ are used and *ℒ* belongs to a class *ℳ*_3_ when *𝒮*_2_, *𝒮*_4_, to *𝒮*_6_ and *𝒮*_11_ to *𝒮*_13_ are used. It is clear that the cause for this difference is the first feature, i.e., (ϰ_1_). The FFNs of ϰ_1_ are (0.21,0.33), (0.22,0.41), (0.11,0.11), (0.33,0.11), and (0.25,0.22) for *ℳ*_1_, *ℳ*_2_, *ℳ*_3_, *ℳ*_4_, and *ℒ*, respectively. It is predicted that the similarity degree between (0.21,0.33) and (0.25,0.22) is larger than the similarity degree between (0.11,0.11) and (0.25,0.22) is larger than the similarity degree between (0.33,0.11) and (0.25,0.22) is larger than (0.22,0.41) and (0.25,0.22). As a conclusion, it appears that *𝒮*(*ℳ*_1_, *ℒ*) > *𝒮*(*ℳ*_3_, *ℒ*) > *𝒮* ( *ℳ*_4_, *ℒ*) > *𝒮*(*ℳ*_2_, *ℒ*) is more acceptable. Similarly, we can find the previously mentioned relations for *𝒮*_2_ to *𝒮*_13_.

## 6. A Comparison of the Proposed Similarity Measures between FFSs

To illustrate the effectiveness of the novel similarity measures for specific FFSs in pattern recognition, we present some examples and compare the novel findings to those reported in the literature.

**Example 6 .**

Comparison analysis of similarity measure for three known patterns *ℳ*_1_, *ℳ*_2_, and *ℳ*_3_ that are presented by the following FFSs in *X*={ϰ_1_, ϰ_2_, ϰ_3_, ϰ_4_}:

 
*ℳ*_1_={(ϰ_1_, 0.3,0.3), (ϰ_2_, 0.4,0.4), (ϰ_3_, 0.4,0.4), (ϰ_4_, 0.4,0.4)},
 
*ℳ*_2_={(ϰ_1_, 0.5,0.5), (ϰ_2_, 0.1,0.1), (ϰ_3_, 0.5,0.5), (ϰ_4_, 0.1,0.1)},
 
*ℳ*_3_={(ϰ_1_, 0.5,0.4), (ϰ_2_, 0.4,0.5), (ϰ_3_, 0.3,0.3), (ϰ_4_, 0.2,0.2)}.

The following is an unknown pattern *ℒ*: *ℒ*={(ϰ_1_, 0.4,0.4), (ϰ_2_, 0.5,0.5), (ϰ_3_, 0.2,0.2), (ϰ_4_, 0.3,0.3)}.

Our objective is to ascertain the class to which *ℒ* belongs. The classification result of the suggested similarity measures (*𝒮*_1_ − *𝒮*_13_) displayed in Table 14 is contrasted to the classification result of the existing similarity measures (*𝒮* [38]-*𝒮* [39]) depicted in Table 15. From Table 14, we observed that the developed similarity measures (*𝒮*_1_, *𝒮*_3_ − *𝒮*_13_) addressing the shortcomings of conventional similarity measures *𝒮* [19], *𝒮* [27], *𝒮* [30], *𝒮* [44], *𝒮* [32], and *𝒮* [45].

**Example 7 .**

Assume that a doctor would like to diagnose the condition of *C*:  (viral fever, malaria, or typhoid) for a set of patients *P*:  (Al, Bob, Joe, and Ted) having symptoms V:  (temperature, headache, and cough). The symptoms associated with the considered diagnosis are listed in Table 16, and the symptoms associated with each patient are listed in Table 17. Each table element is represented by a specific FFSs. Each patient requires proper diagnosis, which need be assessed. We will identify a diagnosis for each patient based on the similarity between the symptoms associated with each diagnosis and those associated with the patient. The diagnostic observations are described in Table 18 Al, Table 19 Bob, Table 20 Joe, and Table 21 Ted, respectively, using the novel similarity measures formula (*𝒮*_1_ − *𝒮*_13_). The patient Al is diagnosed with malaria (Mal.) in 12 of the 13 of the approaches; the remaining approach indicates that Al is diagnosed with viral fever (VF) as presented in Table 18. It is obvious that Bob has a stomach problem (SP), since all of the measures yield the same findings as shown in Table 19. Joe is diagnosed with typhoid in 12 of the 13 methods; the other approach represented that Joe is diagnosed with VF as shown in Table 20. Similarly, 9 of the 13 measures indicated that Ted has VF, whereas, the remaining methods imply that Ted has Mal as presented in Table 21. For patient Al, it could be observed from Table 18 and Table 22 that the established similarity measures (*𝒮*_1_, *𝒮*_3_ − *𝒮*_11_) yield the same findings as those in *𝒮* [41], *𝒮* [10], *𝒮* [49], *𝒮* [13], *𝒮* [12], *𝒮* [44], *𝒮* [32], *𝒮* [48], and *𝒮* [18], and the measures (*𝒮*_2_, *𝒮*_12_, *𝒮*_13_) provide the same results as in *𝒮* [38], *𝒮* [40], *𝒮* [42], *𝒮* [19], *𝒮* [13], *𝒮* [27], *𝒮* [30], *𝒮* [32], *𝒮* [45], *𝒮* [39], *𝒮* [46], *𝒮* [48], *𝒮* [47], and *𝒮* [50]. For patient Bob the novel similarity measures provided the same results as in the literature presented in Table 19 and Table 22. Similarly, for patient Joe the proposed similarity measure provided the same result as in the literature shown in Table 20 except *𝒮*_4_. For patient Ted, the suggested similarity measures (S_1_ − S_6_, S_11_ − S_13_) yield the same findings as in *𝒮* [38], *𝒮* [40], *𝒮* [10], *𝒮* [42], *𝒮* [19], *𝒮* [43], *𝒮* [13], *𝒮* [12], *𝒮* [27], *𝒮* [30], *𝒮* [32], *𝒮* [45], *𝒮* [39], *𝒮* [18], *𝒮* [47], *𝒮* [48], and *𝒮* [50], and the measures S7–S10 provide the results as in *𝒮* [41], *𝒮* [44], *𝒮* [32], *𝒮* [46], and *𝒮* [49] figure out in Tables 21 and 22.  Table 23 shows the present summary of medical diagnosis.

## 7. Application of the Inclusion Measure between FFSs and Pattern Recognition

This section illustrates the applicability of the suggested FFS inclusion measures to pattern recognition.

**Example 8 .**

Let, *ℳ*_1_, *ℳ*_2_, *ℳ*_3_, and *ℳ*_4_ are the known patterns illustrated by FFSs in *X*={ϰ_1_, ϰ_2_, ϰ_3_} described as follows:

 
*ℳ*_1_={(ϰ_1_, 0.21,0.19), (ϰ_2_, 0.22,0.23), (ϰ_3_, 0.32,0.27)},
 
*ℳ*_2_={(ϰ_1_, 0.22,0.12), (ϰ_2_, 0.24,0.21), (ϰ_3_, 0.27,0.25)},
 
*ℳ*_3_={(ϰ_1_, 0.12,0.21), (ϰ_2_, 0.14,0.30), (ϰ_3_, 0.21,0.32)},
 
*ℳ*_4_={(ϰ_1_, 0.10,0.22), (ϰ_2_, 0.11,0.31), (ϰ_3_, 0.13,0.33)}.

The following is an unknown pattern *ℒ*: *ℒ*={(ϰ_1_, 0.15,0.21), (ϰ_2_, 0.19,0.27), and(ϰ_3_, 0.32,0.28)}. Its purpose is to determine which class *ℒ* belongs to. To do this, the inclusion degrees between *ℒ* and classes *ℳ*_1_, *ℳ*_2_, *ℳ*_3_, and *ℳ*_4_ are measured, and *ℒ* is then allocated to the class *ℳ*_*g*_ specified as follows:

(18) $$\begin{array}{c} g=\underset{g}{\mathrm{argmax}}\left\{I\left(M_{g},L\right)\right\}\text{.} \end{array}$$

For all the established inclusion measures (*ℐ*_1_ − *ℐ*_7_) for FFS, the degree of inclusion between *ℐ*(*ℳ*_1_, *ℒ*), *ℐ*(*ℳ*_2_, *ℒ*), *ℐ*(*ℳ*_3_, *ℒ*), and *ℐ*(*ℳ*_4_, *ℒ*) are determined and displayed in Table 24. It is clearly observed in the Table 24 that an unknown pattern *ℒ* belongs to a class *ℳ*_4_ when *ℐ*_1_ to *ℐ*_3_ and *ℐ*_5_ to *ℐ*_6_ are used, and *ℒ* belongs to a class *ℳ*_3_ and *ℳ*_2_ when *ℐ*_4_ and *ℐ*_7_ are, respectively, used. It is clear that the cause for this difference is the first characteristic, i.e., (ϰ_1_). The FFNs of ϰ_1_ are (0.21,0.19), (0.22,0.12), (0.12,0.21), (0.10,0.22), and (0.15,0.21) for *ℳ*_1_, *ℳ*_2_, *ℳ*_3_, *ℳ*_4_, and *ℒ*, respectively. It is predicted that the inclusion degree between (0.10,0.22) and (0.15,0.21) is larger than the inclusion degree between (0.12,0.21) and (0.15,0.21) is larger than the inclusion degree between (0.21,0.19) and (0.15,0.21) is larger than (0.22,0.12) and (0.15,0.21). As a conclusion, it appears that *ℐ*(*ℳ*_1_, *ℒ*) >  *ℐ*(*ℳ*_3_, *ℒ*) > *ℐ*(*ℳ*_4_, *ℒ*) > *ℐ*(*ℳ*_2_, *ℒ*) is more acceptable. In a similar way, we can find the previously mentioned relations for *ℐ*_2_ to *ℐ*_7_.

## 8. Conclusions and Future Recommendations

### 8.1. Conclusions

The main findings of this research are emphasized and encapsulated as follows:

1. We developed axiomatically FFSs information measures (distance measure, similarity measure, entropy, and inclusion measure).
2. We constructed various formulae for FFSs information measures and analyzed the associated transformation relationships in detail.
3. We used the established distance measures (*𝒟*_1_-*𝒟*_13_) to pattern recognition and medical diagnosis to demonstrate their efficacy. The applications substantiate the results and also illustrate the feasibility and effectiveness of the distance measures between FFSs information.
4. We demonstrated the efficacy of the novel similarity measures (S_1_-S_13_); several counterintuitive examples of existing similarity measures are shown. We employed them to pattern recognition, construction materials, and medical diagnosis. For pattern recognition problems, we conclude that the proposed similarity measures dominate existing similarity measures. In some special situations, it has been shown that many conventional similarity measures are incapable of providing reasonable findings. However, in these specific cases, the proposed similarity measure is proficient of discriminating FFSs. A comparison of the proposed similarity measures with conventional similarity measures is performed for medical diagnosis problems. The applications emphasise the results and also illustrate the significance and reliability of the established similarity measures.
5. Additionally, we illustrated the applicability of the suggested FFS inclusion measures to pattern recognition with an example. The findings demonstrate the feasibility and effectiveness of new inclusion measures.

The experimental findings demonstrated that the proposed measures are more reliable and can avoid the counter-intuitive situation in dealing with practical applications based on Fermatean fuzzy environment. [24, 51, 52].

### 8.2. Limitations and Future Works

1. The FFSs are inappropriate to deal with situations where the cube sum of membership and nonmembership grades of exceeds 1
2. A near future target is to unfold the application of the proposed information measures in scientific investigations for decision-making, pattern recognition, linguistic summarization, and data mining
3. We have also a plan to apply the presented approach to procurement planning, water desalination station selection, wind power plant site selection, and many more domains of real world problems
4. Additionally, we will be further interested to immerse them in a variety of fuzzy environments
5. Furthermore, since this work presents an applicative analysis of the FFS information measures, we should develop an appropriate software to effectively apply the presented information measures in a realistic situation

## Figures and Tables

**Table 1:** Distance measures for Example 2with *α*=*β*=0.5, *l*_1_=*l*_2_=2, and *t*=1.

	*𝒟*(*ℳ*_1_, *ℒ*)	*𝒟*(*ℳ*_2_, *ℒ*)	*𝒟*(*ℳ*_3_, *ℒ*)	*𝒟*(*ℳ*_4_, *ℒ*)	Classification results
*𝒟*_1_	0.0827	0.1829	0.4313	0.1137	*ℳ*_1_
*𝒟*_2_	0.0354	0.0504	0.0698	0.0272	*ℳ*_1_
*𝒟*_3_	0.0590	0.1167	0.2505	0.0704	*ℳ*_1_
*𝒟*_4_	0.0514	0.0956	0.2982	0.0638	*ℳ*_1_
*𝒟*_5_	0.0975	0.1738	0.4360	0.1169	*ℳ*_1_
*𝒟*_6_	0.0978	0.1746	0.4593	0.1200	*ℳ*_1_
*𝒟*_7_	0.4118	0.6334	0.7627	0.5214	*ℳ*_1_
*𝒟*_8_	0.7918	0.8745	0.9253	0.8486	*ℳ*_1_
*𝒟*_9_	0.7913	0.8698	0.9226	0.8360	*ℳ*_1_
*𝒟*_10_	0.4141	0.6233	0.7636	0.5184	*ℳ*_1_
*𝒟*_11_	0.6930	0.7167	0.8023	0.6902	*ℳ*_1_
*𝒟*_12_	0.0789	0.1483	0.3761	0.0699	*ℳ*_1_
*𝒟*_13_	0.0316	0.0599	0.1121	0.0212	*ℳ*_1_

**Table 2:** Symptomatic characteristics of the diagnosis under consideration.

	Headache	Acidity	Burning eyes	Depression
Stress	(0.2,0.6)	(0.3,0.7)	(0.2,0.6)	(0.1,0.8)
Ulcer	(0.3,0.7)	(0.1,0.4)	(0.2,0.4)	(0.1,0.4)
Vision problem	(0.2,0.5)	(0.1,0.5)	(0.1,0.3)	(0.2,0.3)
Blood pressure	(0.1,0.4)	(0.2,0.5)	(0.2,0.4)	(0.1,0.3)

**Table 3:** Symptoms and features of the patients under consideration.

	Blood pressure	Ulcer	Vision problem	Stress
Ragu	(0.2,0.4)	(0.1,0.3)	(0.1,0.4)	(0.2,0.3)
Mathi	(0.1,0.5)	(0.1,0.4)	(0.2,0.3)	(0.1,0.3)
Velu	(0.1,0.3)	(0.1,0.5)	(0.2,0.3)	(0.2,0.3)
Karthi	(0.1,0.4)	(0.2,0.4)	(0.1,0.5)	(0.1,0.3)

**Table 4:** The distance between the patient and the set of probable diagnoses using *𝒟*_1_.

	Blood pressure	Ulcer	Vision problem	Stress
Ragu	0.2961	0.1539	0.1047	0.0760
Mathi	0.2658	0.1236	0.0459	0.0899
Velu	0.2882	0.2062	0.0683	0.0522
Karthi	0.2536	0.1618	0.1093	0.0803

**Table 5:** Symptomatic characteristics of the patient.

	ϰ_1_	ϰ_2_	ϰ_3_	ϰ_4_	ϰ_5_
*ℳ*_1_	(0.80,0.10)	(0.60,0.10)	(0.20,0.80)	(0.60,0.10)	(0.10,0.60)
*ℳ*_2_	(0.00,0.80)	(0.40,0.40)	(0.60,0.10)	(0.10,0.70)	(0.10,0.80)
*ℳ*_3_	(0.80,0.10)	(0.80,0.10)	(0.00,0.60)	(0.20,0.70)	(0.00,0.50)
*ℳ*_4_	(0.60,0.10)	(0.50,0.40)	(0.30,0.40)	(0.70,0.20)	(0.30,0.40)

**Table 6:** Symptomatic characteristics of the diagnosis.

	ϰ_1_	ϰ_2_	ϰ_3_	ϰ_4_	ϰ_5_
*ℒ*_1_	(0.40,0.00)	(0.30,0.50)	(0.10,0.70)	(0.40,0.30)	(0.10,0.70)
*ℒ*_2_	(0.70,0.00)	(0.20,0.60)	(0.00,0.90)	(0.70,0.00)	(0.10,0.80)
*ℒ*_3_	(0.30,0.30)	(0.60,0.10)	(0.20,0.70)	(0.20,0.60)	(0.10,0.90)
*ℒ*_4_	(0.10,0.70)	(0.20,0.40)	(0.80,0.00)	(0.20,0.70)	(0.20,0.70)
*ℒ*_5_	(0.10,0.80)	(0.00,0.80)	(0.20,0.80)	(0.20,0.80)	(0.80,0.10)

**Table 7:** Diagnostic results of the FFS distance measure for Al.

	*𝒟*(*ℳ*_1_, *ℒ*_1_)	*𝒟*(*ℳ*_1_, *ℒ*_2_)	*𝒟*(*ℳ*_1_, *ℒ*_3_)	*𝒟*(*ℳ*_1_, *ℒ*_4_)	*𝒟*(*ℳ*_1_, *ℒ*_5_)	Classification results
*𝒟*_1_	0.3643	0.3417	0.4607	0.5690	0.6813	*ℒ*_2_
*𝒟*_2_	0.2045	0.2067	0.2693	0.4683	0.5323	*ℒ*_1_
*𝒟*_3_	0.2844	0.2742	0.3650	0.5187	0.6068	*ℒ*_2_
*𝒟*_4_	0.3617	0.3413	0.4607	0.5667	0.6813	*ℒ*_2_
*𝒟*_5_	0.5717	0.5606	0.6581	0.8110	0.9018	*ℒ*_2_
*𝒟*_6_	0.3566	0.3400	0.4331	0.5075	0.5804	*ℒ*_2_
*𝒟*_7_	0.6394	0.4872	0.6744	0.9229	0.8799	*ℒ*_2_
*𝒟*_8_	0.8703	0.8057	0.9047	0.9777	0.9591	*ℒ*_2_
*𝒟*_9_	0.8744	0.8199	0.8978	0.9864	0.9678	*ℒ*_2_
*𝒟*_1_	0.6339	0.4892	0.6628	0.9202	0.8568	*ℒ*_2_
*𝒟*_11_	0.7457	0.7334	0.7621	0.8274	0.8555	*ℒ*_2_
*𝒟*_12_	0.2305	0.2321	0.2998	0.4522	0.5370	*ℒ*_2_
*𝒟*_13_	0.2146	0.1679	0.2480	0.3912	0.4480	*ℒ*_2_

**Table 8:** Diagnostic results of the FFS distance measure for Bob.

	*𝒟*(*ℳ*_2_, *ℒ*_1_)	*𝒟*(*ℳ*_2_, *ℒ*_2_)	*𝒟*(*ℳ*_2_, *ℒ*_3_)	*𝒟*(*ℳ*_2_, *ℒ*_4_)	*𝒟*(*ℳ*_2_, *ℒ*_5_)	Classification results
*𝒟*_1_	0.4667	0.5783	0.4410	0.2323	0.5490	*ℒ*_4_
*𝒟*_2_	0.2965	0.4487	0.2713	0.1177	0.4027	*ℒ*_4_
*𝒟*_3_	0.3816	0.5135	0.3562	0.1750	0.4758	*ℒ*_4_
*𝒟*_4_	0.4667	0.5783	0.4410	0.2323	0.5467	*ℒ*_4_
*𝒟*_5_	0.6904	0.7648	0.6696	0.3850	0.7542	*ℒ*_4_
*𝒟*_6_	0.4375	0.5152	0.4185	0.2447	0.4940	*ℒ*_4_
*𝒟*_7_	0.8448	0.8700	0.7308	0.4278	0.8139	*ℒ*_4_
*𝒟*_8_	0.9528	0.9617	0.8980	0.8085	0.9388	*ℒ*_4_
*𝒟*_9_	0.9380	0.9610	0.8783	0.7880	0.9336	*ℒ*_4_
*𝒟*_10_	0.7928	0.8210	0.6621	0.3486	0.7230	*ℒ*_4_
*𝒟*_11_	0.7899	0.8471	0.7811	0.7201	0.8534	*ℒ*_4_
*𝒟*_12_	0.3577	0.4994	0.3456	0.1569	0.4980	*ℒ*_4_
*𝒟*_13_	0.2854	0.3870	0.2447	0.1432	0.3383	*ℒ*_4_

**Table 9:** Diagnostic results of the FFS distance measure for Joe.

	*𝒟*(*ℳ*_3_, *ℒ*_1_)	*𝒟*(*ℳ*_3_, *ℒ*_2_)	*𝒟*(*ℳ*_3_, *ℒ*_3_)	*𝒟*(*ℳ*_3_, *ℒ*_4_)	*𝒟*(*ℳ*_3_, *ℒ*_5_)	Classification results
*𝒟*_1_	0.5323	0.6393	0.5493	0.5843	0.6693	*ℒ*_3_
*𝒟*_2_	0.2952	0.4107	0.2760	0.3930	0.5230	*ℒ*_3_
*𝒟*_3_	0.4137	0.5250	0.4127	0.4887	0.5962	*ℒ*_3_
*𝒟*_4_	0.5313	0.6387	0.5463	0.5817	0.6667	*ℒ*_1_
*𝒟*_5_	0.7785	0.9021	0.7713	0.7939	0.9256	*ℒ*_3_
*𝒟*_6_	0.4835	0.5541	0.4937	0.5174	0.5714	*ℒ*_1_
*𝒟*_7_	0.7948	0.7738	0.6857	0.8150	0.8678	*ℒ*_3_
*𝒟*_8_	0.9280	0.9141	0.9085	0.9326	0.9549	*ℒ*_3_
*𝒟*_9_	0.9433	0.9337	0.9070	0.9685	0.9673	*ℒ*_3_
*𝒟*_10_	0.7915	0.7788	0.6739	0.8298	0.8467	*ℒ*_3_
*𝒟*_11_	0.7747	0.7954	0.7657	0.8089	0.8521	*ℒ*_3_
*𝒟*_12_	0.3106	0.4096	0.3054	0.3903	0.5265	*ℒ*_3_
*𝒟*_13_	0.2632	0.3302	0.2512	0.3433	0.4011	*ℒ*_3_

**Table 10:** Diagnostic results of the FFS distance measure for Ted.

	*𝒟*(*ℳ*_4_, *ℒ*_1_)	*𝒟*(*ℳ*_4_, *ℒ*_2_)	*𝒟*(*ℳ*_4_, *ℒ*_3_)	*𝒟*(*ℳ*_4_, *ℒ*_4_)	*𝒟*(*ℳ*_4_, *ℒ*_5_)	Classification results
*𝒟*_1_	0.3627	0.4667	0.5197	0.5193	0.7987	*ℒ*_1_
*𝒟*_2_	0.2030	0.2618	0.3168	0.3652	0.5255	*ℒ*_1_
*𝒟*_3_	0.2828	0.3643	0.4183	0.4423	0.6621	*ℒ*_1_
*𝒟*_4_	0.3623	0.4667	0.5197	0.5193	0.7987	*ℒ*_1_
*𝒟*_5_	0.5837	0.6409	0.7406	0.7702	0.0791	*ℒ*_1_
*𝒟*_6_	0.3572	0.4375	0.4754	0.4751	0.6479	*ℒ*_1_
*𝒟*_7_	0.7744	0.6062	0.8480	0.9195	0.9493	*ℒ*_2_
*𝒟*_8_	0.9295	0.8542	0.9551	0.9798	0.9825	*ℒ*_2_
*𝒟*_9_	0.9286	0.9173	0.9635	0.9824	0.9839	*ℒ*_2_
*𝒟*_10_	0.7736	0.6740	0.8610	0.9206	0.9454	*ℒ*_2_
*𝒟*_11_	0.7382	0.7472	0.7742	0.7901	0.8373	*ℒ*_1_
*𝒟*_12_	0.2203	0.2769	0.3340	0.3600	0.5182	*ℒ*_1_
*𝒟*_13_	0.1652	0.1903	0.2343	0.2774	0.3843	*ℒ*_1_

**Table 11:** Diagnosis results of different methods in Example 4 (summary of the comparison).

	Al	Bob	Joe	Ted
Wei et al. [18]	Mal	SP	TYP	VF
Xiao and Ding [35]	Mal	SP	TYP	VF
Zhou et al. [36]	Mal	SP	TYP	VF
Euclidean distance	Mal	SP	TYP	VF
Deng and Wang [37]	Mal	SP	TYP	VF
Deng and Wang [37]	Mal	SP	TYP	VF
Present	—	—	—	—
*𝒟*_1_	Mal	SP	TYP	VF
*𝒟*_2_	**VF**	SP	TYP	VF
*𝒟*_3_	Mal	SP	TYP	VF
*𝒟*_4_	Mal	SP	**VF**	VF
*𝒟*_5_	Mal	SP	TYP	VF
*𝒟*_6_	Mal	SP	**VF**	VF
*𝒟*_7_	Mal	SP	TYP	VF
*𝒟*_8_	Mal	SP	TYP	VF
*𝒟*_9_	Mal	SP	TYP	VF
*𝒟*_10_	Mal	SP	TYP	VF
*𝒟*_11_	Mal	SP	TYP	VF
*𝒟*_12_	Mal	SP	TYP	VF
*𝒟*_13_	Mal	SP	TYP	VF

**Table 12:** Classes information with FFSs.

	ϰ_1_	ϰ_2_	ϰ_3_
*ℳ*_1_	(0.21,0.33)	(0.22,0.43)	(0.32,0.32)
*ℳ*_2_	(0.22,0.41)	(0.32,0.31)	(0.13,0.34)
*ℳ*_3_	(0.11,0.11)	(0.12,0.21)	(0.32,0.32)
*ℳ*_4_	(0.33,0.11)	(0.21,0.22)	(0.23,0.32)
*ℒ*	(0.25,0.22)	(0.42,0.34)	(0.33,0.24)

**Table 13:** Similarity measure for Example 5.

	*𝒮*(*ℳ*_1_, *ℒ*)	*𝒮*(*ℳ*_2_, *ℒ*)	*𝒮*(*ℳ*_3_, *ℒ*)	*𝒮*(*ℳ*_4_, *ℒ*)	Classification results
*𝒮*_1_	0.9641	0.9524	0.9517	0.9541	*ℳ*_1_
*𝒮*_2_	0.9738	0.9743	0.9884	0.9819	*ℳ*_3_
*𝒮*_3_	0.9689	0.9633	0.9701	0.9680	*ℳ*_1_
*𝒮*_4_	0.9731	0.9667	0.9736	0.9728	*ℳ*_3_
*𝒮*_5_	0.1636	0.1629	0.1637	0.1636	*ℳ*_3_
*𝒮*_6_	0.4909	0.4886	0.4910	0.4908	*ℳ*_3_
*𝒮*_7_	0.4248	0.3654	0.2901	0.2829	*ℳ*_1_
*𝒮*_8_	0.1261	0.1155	0.0822	0.0888	*ℳ*_1_
*𝒮*_9_	0.1281	0.1312	0.0824	0.0816	*ℳ*_2_
*𝒮*_10_	0.4252	0.3656	0.2891	0.2749	*ℳ*_1_
*𝒮*_11_	0.3161	0.3146	0.3172	0.3155	*ℳ*_3_
*𝒮*_12_	0.9486	0.9436	0.9522	0.9469	*ℳ*_3_
*𝒮*_13_	0.9760	0.9776	0.9907	0.9848	*ℳ*_3_

**Table 14:** Comparison for the proposed similarity measure for Example 6.

	*𝒮*(*ℳ*_1_, *ℒ*)	*𝒮*(*ℳ*_2_, *ℒ*)	*𝒮*(*ℳ*_3_, *ℒ*)	Classification result
*𝒮*_1_	0.8973	0.7987	0.9467	*ℳ*_3_
*𝒮*_2_	1.0000	1.0000	0.9797	Cannot be classified
*𝒮*_3_	0.9487	0.8993	0.9632	*ℳ*_3_
*𝒮*_4_	0.9615	0.9245	0.9648	*ℳ*_3_
*𝒮*_5_	0.1623	0.1579	0.1627	*ℳ*_3_
*𝒮*_6_	0.4868	0.4735	0.4880	*ℳ*_3_
*𝒮*_7_	0.3913	0.1947	0.7015	*ℳ*_3_
*𝒮*_8_	0.1433	0.0649	0.2470	*ℳ*_3_
*𝒮*_9_	0.1433	0.0649	0.2383	*ℳ*_3_
*𝒮*_10_	0.3913	0.1947	0.6755	*ℳ*_3_
*𝒮*_11_	0.3007	0.2720	0.3160	*ℳ*_3_
*𝒮*_12_	0.9023	0.8171	0.9481	*ℳ*_3_
*𝒮*_13_	0.9828	0.9721	0.9760	*ℳ*_1_

**Table 15:** Comparison for similarity measures for Example 6.

	*𝒮*(*ℳ*_1_, *ℒ*)	*𝒮*(*ℳ*_2_, *ℒ*)	*𝒮*(*ℳ*_3_, *ℒ*)	Classification results
*𝒮* [38]	0.8677	0.7261	0.9134	*ℳ*_3_
*𝒮* [40]	1.0000	1.0000	0.9750	Cannot be classified
*𝒮* [41]	0.8679	0.7425	0.8923	*ℳ*_3_
*𝒮* [10]	0.8750	0.7500	0.9000	*ℳ*_3_
*𝒮* [10]	0.8141	0.6501	0.8495	*ℳ*_3_
*𝒮* [10]	0.7778	0.6000	0.8182	*ℳ*_3_
*𝒮* [42]	0.8750	0.7500	0.9250	*ℳ*_3_
*𝒮* [19]	1.0000	1.0000	0.9750	Cannot be classified
*𝒮* [43]	0.9375	0.8750	0.9500	*ℳ*_3_
*𝒮* [13]	0.8750	0.7500	0.9250	*ℳ*_3_
*𝒮* [13]	0.9375	0.8750	0.9500	*ℳ*_3_
*𝒮* [13]	0.9167	0.8333	0.9417	*ℳ*_3_
*𝒮* [12]	0.8750	0.7500	0.9250	*ℳ*_3_
*𝒮* [27]	1.0000	1.0000	0.9969	Cannot be classified
*𝒮* [30]	1.0000	1.0000	0.9884	Cannot be classified
*𝒮* [44]	0.5000	0.5000	0.5000	Cannot be classified
*𝒮* [32]	1.0000	1.0000	0.9775	Cannot be classified
*𝒮* [32]	0.5038	0.2378	0.6223	*ℳ*_3_
*𝒮* [32]	0.8785	0.7912	0.9205	*ℳ*_3_
*𝒮* [45]	0.9583	0.9167	0.9583	Cannot be classified
*𝒮* [39]	0.9841	0.9727	0.9816	*ℳ*_1_

**Table 16:** Symptom features for the diagnosis under consideration.

	Temperature	Headache	Stomach pain	Cough	Chest pain
Viral fever	(0.4,0.0)	(0.3,0.5)	(0.1,0.7)	(0.4,0.3)	(0.1,0.7)
Malaria	(0.7,0.0)	(0.2,0.6)	(0.0,0.9)	(0.7,0.0)	(0.1,0.8)
Typhoid	(0.3,0.3)	(0.6,0.1)	(0.2,0.7)	(0.2,0.6)	(0.1,0.9)
Stomach problem	(0.1,0.7)	(0.2,0.4)	(0.8,0.0)	(0.2,0.7)	(0.2,0.7)
Chest problem	(0.1,0.8)	(0.0,0.8)	(0.2,0.8)	(0.2,0.8)	(0.8,0.1)

**Table 17:** Symptoms of the patients under examination.

	Temperature	Headache	Stomach pain	Cough	Chest pain
Al	(0.8,0.1)	(0.6,0.1)	(0.2,0.8)	(0.6,0.1)	0.1, 0.6
Bob	(0.0,0.8)	(0.4,0.4)	(0.6,0.1)	(0.1,0.7)	0.1, 0.8
Joe	(0.8,0.1)	(0.8,0.1)	(0.0,0.6)	(0.2,0.7)	0.0, 0.5
Ted	(0.6,0.1)	(0.5,0.4)	(0.3,0.4)	(0.7,0.2)	0.3, 0.4

**Table 18:** Symptoms features of the patient (Al).

	VF	Mal	Typ	SP	Chest problem
*𝒮*_1_	0.6488	0.6984	0.7203	0.5014	0.4562
*𝒮*_2_	0.7955	0.7933	0.7307	0.5317	0.4677
*𝒮*_3_	0.7221	0.7459	0.7255	0.5165	0.4619
*𝒮*_4_	0.7288	0.7440	0.6545	0.5750	0.4890
*𝒮*_5_	0.1472	0.1478	0.1424	0.1343	0.1272
*𝒮*_6_	0.4391	0.4430	0.4198	0.3976	0.3715
*𝒮*_7_	0.3606	0.5128	0.3256	0.0771	0.1201
*𝒮*_8_	0.1297	0.1943	0.0953	0.0223	0.0409
*𝒮*_9_	0.1256	0.1801	0.1022	0.0136	0.0322
*𝒮*_10_	0.3661	0.5108	0.3372	0.0798	0.1432
*𝒮*_11_	0.2543	0.2666	0.2379	0.1726	0.1445
*𝒮*_12_	0.7695	0.7679	0.7002	0.5478	0.4630
*𝒮*_13_	0.8437	0.7623	0.7557	0.6000	0.5030

**Table 19:** Symptoms features of the patient (Bob).

	VF	Mal	Typ	SP	Chest problem
*𝒮*_1_	0.5582	0.4509	0.7143	0.8666	0.5881
*𝒮*_2_	0.7035	0.5513	0.7287	0.8823	0.5973
*𝒮*_3_	0.6309	0.5011	0.7215	0.8745	0.5927
*𝒮*_4_	0.6500	0.5662	0.6693	0.8257	0.5900
*𝒮*_5_	0.1411	0.1364	0.1424	0.1547	0.1370
*𝒮*_6_	0.4186	0.3950	0.4238	0.4625	0.4019
*𝒮*_7_	0.1552	0.1300	0.2692	0.5722	0.1861
*𝒮*_8_	0.0472	0.0383	0.1020	0.1915	0.0612
*𝒮*_9_	0.0620	0.0390	0.1217	0.2120	0.0664
*𝒮*_10_	0.2072	0.1790	0.3379	0.6514	0.2770
*𝒮*_11_	0.2101	0.1529	0.2189	0.2799	0.1466
*𝒮*_12_	0.6423	0.5006	0.6544	0.8431	0.5020
*𝒮*_13_	0.7719	0.5822	0.7506	0.8711	0.5903

**Table 20:** Symptoms features of the patient (Joe).

	VF	Mal	Typ	SP	Chest problem
*𝒮*_1_	0.5616	0.4917	0.7086	0.5813	0.4720
*𝒮*_2_	0.7048	0.5893	0.7240	0.6070	0.4770
*𝒮*_3_	0.6332	0.5405	0.7163	0.5942	0.4745
*𝒮*_4_	0.6015	0.5210	0.5902	0.5637	0.5000
*𝒮*_5_	0.1363	0.1284	0.1366	0.1346	0.1266
*𝒮*_6_	0.4052	0.3815	0.4020	0.3943	0.3750
*𝒮*_7_	0.2052	0.2262	0.3143	0.1850	0.1322
*𝒮*_8_	0.0720	0.0859	0.0915	0.0674	0.0451
*𝒮*_9_	0.0567	0.0663	0.0930	0.0315	0.0327
*𝒮*_10_	0.2085	0.2212	0.3261	0.1702	0.1533
*𝒮*_11_	0.2253	0.2046	0.2343	0.1911	0.1479
*𝒮*_12_	0.6894	0.5904	0.6946	0.6097	0.4735
*𝒮*_13_	0.7613	0.6263	0.7706	0.6493	0.5101

**Table 21:** Symptoms features of the patient (Ted).

	VF	Mal	Typ	SP	Chest problem
*𝒮*_1_	0.6579	0.6447	0.6727	0.6080	0.4644
*𝒮*_2_	0.7970	0.7382	0.6832	0.6348	0.4745
*𝒮*_3_	0.7274	0.6914	0.6779	0.6214	0.4694
*𝒮*_4_	0.7283	0.6500	0.6103	0.6105	0.4010
*𝒮*_5_	0.1467	0.1433	0.1385	0.1369	0.1142
*𝒮*_6_	0.4390	0.4186	0.4077	0.4077	0.3425
*𝒮*_7_	0.2256	0.3938	0.1520	0.0805	0.0507
*𝒮*_8_	0.0705	0.1458	0.0449	0.0202	0.0175
*𝒮*_9_	0.0714	0.0827	0.0365	0.0176	0.0161
*𝒮*_10_	0.2264	0.3260	0.1390	0.0794	0.0546
*𝒮*_11_	0.2618	0.2528	0.2258	0.2099	0.1627
*𝒮*_12_	0.7797	0.7231	0.6660	0.6400	0.4818
*𝒮*_13_	0.8268	0.7069	0.7062	0.6688	0.5084

**Table 22:** The summary of existing similarity measures in medical diagnosis.

	Al	Bob	Joe	Ted
*𝒮* [38]	VF	SP	TYP	VF
*𝒮* [40]	VF	SP	TYP	VF
*𝒮* [41]	Mal	SP	TYP	Mal
*𝒮* [10]	Mal	SP	TYP	VF
*𝒮* [10]	Mal	SP	TYP	VF
*𝒮* [10]	Mal	SP	TYP	VF
*𝒮* [42]	VF	SP	TYP	VF
*𝒮* [19]	VF	SP	TYP	VF
*𝒮* [43]	VF/Mal	SP	TYP	VF
*𝒮* [13]	Mal	SP	TYP	VF
*𝒮* [13]	VF	SP	TYP	VF
*𝒮* [13]	Mal	SP	TYP	VF
*𝒮* [12]	Mal	SP	TYP	VF
*𝒮* [27]	VF	SP	TYP	VF
*𝒮* [30]	VF	SP	TYP	VF
*𝒮* [44]	Mal	SP	TYP	Mal
*𝒮* [32]	Mal	SP	TYP	VF
*𝒮* [32]	Mal	SP	TYP	Mal
*𝒮* [32]	VF	SP	TYP	VF
*𝒮* [45]	VF	SP	TYP	VF
*𝒮* [39]	VF	SP	TYP	VF
*𝒮* [18]	Mal	SP	TYP	VF
*𝒮* [46]	VF	SP	TYP	Mal
*𝒮* [47]	VF	SP	TYP	VF
*𝒮* [48]	VF	SP	TYP	VF
*𝒮* [48]	VF	SP	TYP	VF
*𝒮* [48]	Mal	SP	SP	VF
*𝒮* [49]	Mal	SP	TYP	Mal
*𝒮* [50]	VF	SP	TYP	VF

**Table 23:** The summary of present similarity measures in medical diagnosis.

	Al	Bob	Joe	Ted
*𝒮*_1_	TYP	SP	TYP	TYP
*𝒮*_2_	VF	SP	TYP	VF
*𝒮*_3_	Mal	SP	TYP	VF
*𝒮*_4_	Mal	SP	VF	VF
*𝒮*_5_	Mal	SP	TYP	VF
*𝒮*_6_	Mal	SP	VF	VF
*𝒮*_7_	Mal	SP	TYP	Mal
*𝒮*_8_	Mal	SP	TYP	Mal
*𝒮*_9_	Mal	SP	TYP	Mal
*𝒮*_10_	Mal	SP	TYP	Mal
*𝒮*_11_	Mal	SP	TYP	VF
*𝒮*_12_	VF	SP	TYP	VF
*𝒮*_13_	VF	SP	TYP	VF

**Table 24:** Inclusion measure for Example 8.

	*ℐ*(*ℳ*_1_, *ℒ*)	*ℐ*(*ℳ*_2_, *ℒ*)	*ℐ*(*ℳ*_3_, *ℒ*)	*ℐ*(*ℳ*_4_, *ℒ*)	Classification results
*ℐ*_1_	0.9964	0.9936	0.9970	**0.9997**	*ℳ*_4_
*ℐ*_2_	0.9927	0.9872	0.9939	**0.9995**	*ℳ*_4_
*ℐ*_3_	0.9927	0.9873	0.9940	**0.9995**	*ℳ*_4_
*ℐ*_4_	0.9732	0.9787	**0.9906**	0.9852	*ℳ*_3_
*ℐ*_5_	0.3309	0.3291	0.3313	**0.3332**	*ℳ*_4_
*ℐ*_6_	0.3309	0.3291	0.3313	**0.3332**	*ℳ*_4_
*ℐ*_7_	0.3356	**0.3369**	0.3331	0.3321	*ℳ*_2_

## Data Availability

The data used in this manuscript are hypothetical and can be used by anyone by just citing this article.
